# Integrative study of lung cancer adeno-to-squamous transition in EGFR TKI resistance identifies RAPGEF3 as a therapeutic target

**DOI:** 10.1093/nsr/nwae392

**Published:** 2024-11-07

**Authors:** Hua Wang, Shijie Tang, Qibiao Wu, Yayi He, Weikang Zhu, Xinyun Xie, Zhen Qin, Xue Wang, Shiyu Zhou, Shun Yao, Xiaoling Xu, Chenchen Guo, Xinyuan Tong, Shuo Han, Yueh-Hung Chou, Yong Wang, Kwok-Kin Wong, Cai-Guang Yang, Luonan Chen, Liang Hu, Hongbin Ji

**Affiliations:** Key Laboratory of Multi-Cell Systems, Shanghai Institute of Biochemistry and Cell Biology, Center for Excellence in Molecular Cell Science, Chinese Academy of Sciences, Shanghai 200031, China; University of Chinese Academy of Sciences, Beijing 100049, China; Key Laboratory of Multi-Cell Systems, Shanghai Institute of Biochemistry and Cell Biology, Center for Excellence in Molecular Cell Science, Chinese Academy of Sciences, Shanghai 200031, China; Key Laboratory of Multi-Cell Systems, Shanghai Institute of Biochemistry and Cell Biology, Center for Excellence in Molecular Cell Science, Chinese Academy of Sciences, Shanghai 200031, China; University of Chinese Academy of Sciences, Beijing 100049, China; Department of Medical Oncology, Shanghai Pulmonary Hospital, Tongji University Medical School Cancer Institute, Tongji University School of Medicine, Shanghai 200092, China; Center for Excellence in Mathematical Sciences, National Center for Mathematics and Interdisciplinary Sciences, Key Laboratory of Management, Decision and Information System, Hua Loo-Keng Center for Mathematical Sciences, Academy of Mathematics and Systems Science, Chinese Academy of Sciences, Beijing 100190, China; University of Chinese Academy of Sciences, Beijing 100049, China; State Key Laboratory of Drug Research, Shanghai Institute of Materia Medica, Chinese Academy of Sciences, Shanghai 201203, China; School of Pharmaceutical Science and Technology, Hangzhou Institute for Advanced Study, University of Chinese Academy of Sciences, Hangzhou 310024, China; Key Laboratory of Multi-Cell Systems, Shanghai Institute of Biochemistry and Cell Biology, Center for Excellence in Molecular Cell Science, Chinese Academy of Sciences, Shanghai 200031, China; Key Laboratory of Multi-Cell Systems, Shanghai Institute of Biochemistry and Cell Biology, Center for Excellence in Molecular Cell Science, Chinese Academy of Sciences, Shanghai 200031, China; Key Laboratory of Multi-Cell Systems, Shanghai Institute of Biochemistry and Cell Biology, Center for Excellence in Molecular Cell Science, Chinese Academy of Sciences, Shanghai 200031, China; Key Laboratory of Multi-Cell Systems, Shanghai Institute of Biochemistry and Cell Biology, Center for Excellence in Molecular Cell Science, Chinese Academy of Sciences, Shanghai 200031, China; Department of Radiation Oncology, Shanghai Pulmonary Hospital, Tongji University School of Medicine, Shanghai 200433, China; Key Laboratory of Multi-Cell Systems, Shanghai Institute of Biochemistry and Cell Biology, Center for Excellence in Molecular Cell Science, Chinese Academy of Sciences, Shanghai 200031, China; Key Laboratory of Multi-Cell Systems, Shanghai Institute of Biochemistry and Cell Biology, Center for Excellence in Molecular Cell Science, Chinese Academy of Sciences, Shanghai 200031, China; Key Laboratory of Multi-Cell Systems, Shanghai Institute of Biochemistry and Cell Biology, Center for Excellence in Molecular Cell Science, Chinese Academy of Sciences, Shanghai 200031, China; Department of Anatomical Pathology, Far Eastern Memorial Hospital, New Taipei City, Taiwan, China; Center for Excellence in Mathematical Sciences, National Center for Mathematics and Interdisciplinary Sciences, Key Laboratory of Management, Decision and Information System, Hua Loo-Keng Center for Mathematical Sciences, Academy of Mathematics and Systems Science, Chinese Academy of Sciences, Beijing 100190, China; Laura and Isaac Perlmutter Cancer Center, New York University Grossman School of Medicine, NYU Langone Health, NY 10016, USA; University of Chinese Academy of Sciences, Beijing 100049, China; State Key Laboratory of Drug Research, Shanghai Institute of Materia Medica, Chinese Academy of Sciences, Shanghai 201203, China; School of Pharmaceutical Science and Technology, Hangzhou Institute for Advanced Study, University of Chinese Academy of Sciences, Hangzhou 310024, China; Key Laboratory of Multi-Cell Systems, Shanghai Institute of Biochemistry and Cell Biology, Center for Excellence in Molecular Cell Science, Chinese Academy of Sciences, Shanghai 200031, China; University of Chinese Academy of Sciences, Beijing 100049, China; School of Life Science and Technology, Shanghai Tech University, Shanghai 201210, China; School of Life Science, Hangzhou Institute for Advanced Study, University of Chinese Academy of Sciences, Hangzhou 310024, China; Key Laboratory of Multi-Cell Systems, Shanghai Institute of Biochemistry and Cell Biology, Center for Excellence in Molecular Cell Science, Chinese Academy of Sciences, Shanghai 200031, China; Key Laboratory of Multi-Cell Systems, Shanghai Institute of Biochemistry and Cell Biology, Center for Excellence in Molecular Cell Science, Chinese Academy of Sciences, Shanghai 200031, China; University of Chinese Academy of Sciences, Beijing 100049, China; School of Life Science and Technology, Shanghai Tech University, Shanghai 201210, China; School of Life Science, Hangzhou Institute for Advanced Study, University of Chinese Academy of Sciences, Hangzhou 310024, China

**Keywords:** EGFR-mutant lung cancer, adeno-to-squamous transition, tyrosine kinase inhibitor resistance, transcription factor network, RAPGEF3

## Abstract

Although adeno-to-squamous transition (AST) has been observed in association with resistance to epidermal growth factor receptor (EGFR) tyrosine kinase inhibitor (TKI) in clinic, its causality, molecular mechanism and overcoming strategies remain largely unclear. We here demonstrate that squamous transition occurs concomitantly with TKI resistance in PC9-derived xenograft tumors. Perturbation of squamous transition via DNp63 overexpression or knockdown leads to significant changes in TKI responses, indicative of a direct causal link between squamous transition and TKI resistance. Integrative RNA-seq, ATAC-seq analyses and functional studies reveal that FOXA1 plays an important role in maintaining adenomatous lineage and contributes to TKI sensitivity. FOXM1 overexpression together with FOXA1 knockout fully recapitulates squamous transition and TKI resistance in both PC9 xenografts and patient-derived xenograft (PDX) models. Importantly, pharmacological inhibition of RAPGEF3 combined with EGFR TKI efficiently overcomes TKI resistance, especially in RAPGEF3^high^ PDXs. Our findings provide novel mechanistic insights into squamous transition and therapeutic strategy to overcome EGFR TKI resistance in lung cancer.

## INTRODUCTION

Lung cancer is one of the deadliest diseases worldwide, and lung adenocarcinoma (ADC) and lung squamous cell carcinoma (SCC) are two major subtypes with distinct molecular, histological and clinical features. Epidermal growth factor receptor (EGFR) mutations are well established as an important oncogenic driver in lung ADC, especially in female, non-smokers and Eastern Asian populations [1]. Patients with EGFR-mutant lung ADC initially respond to EGFR tyrosine kinase inhibitors (TKIs) including gefitinib, erlotinib or osimertinib quite well [2–4]. However, drug resistance occurs inevitably after a median of 10–19 months [5]. Multiple EGFR TKI resistant mechanisms have been documented, e.g. the appearance of new EGFR mutations including T790M or C797S mutations [6,7], the activation of a bypass signaling pathway [8,9], epithelial-mesenchymal transition (EMT) or histological transition to small cell lung cancer (SCLC) [10,11]. Recently, adeno-to-squamous transition (AST) has been observed in EGFR-mutant lung ADC patients relapsed from TKI treatments [12,13]. Clinical data also show that ∼7%–9% patients relapsed from first-line or later-line osimertinib therapy exhibit squamous transition [14]. Although the causality between AST and TKI resistance remains unclear, these observations implicate a potential link between these two biological events.

It is well established that lung ADCs and SCCs are two major subtypes of non-small cell lung cancer (NSCLC) with distinct pathological and molecular features. Lung ADC usually displays glandular features with acini, tubules, or papillary structures [15], and express adenomatous lineage-specific markers including transcription factor (TF) NKX2.1 [16]. In contrast, lung SCC tends to exhibit prominent intercellular bridge and keratinization, and express squamous lineage genes including DNp63, KRT5 and KRT14 [16–19]. Through the integrative studies of 109 human lung adenosquamous carcinoma specimens using WGS and RNA-seq, we have recently proposed a model for dynamic progressive squamous transition via the dysregulation of the adenomatous-specific TFs (NKX2.1 and FOXA2) and squamous-specific TFs (p63 and SOX2) [20], e.g. the terminal respiratory unit (TRU)-like subtype with dominant activities of two adenomatous-lineage TFs (NKX2.1 and FOXA2) gradually decreases, and through an intermediate stage with high inflammation, the cancer eventually transitions to basal-like subtype with high activities of squamous-lineage TFs (p63 and SOX2) [20]. Consistent with these clinical findings, simultaneous knockouts of *Nkx2.1*, *FoxA1* and *FoxA2* have been shown to drive squamous transition of *Kras^G12D^* lung ADC in mice [21]. Moreover, a recent study of pre- and post-transition clinical specimens has uncovered an importance of epigenetic regulation and transcriptional reprogramming involving AKT, MYC and PRC2 signaling [22]. These studies have begun to shed light on the molecular mechanisms of AST and its potential link to clinical therapeutic failure.

Through integrative analyses of human EGFR-mutant lung cancer cell-derived xenografts, PDX models and clinical specimens, we here provide evidence to support a causal link between AST and TKI resistance, reveal the underlying molecular mechanism and offer a potential therapeutic strategy to overcome AST-mediated drug resistance in lung cancer.

## RESULTS

### Squamous transition occurs concomitantly with TKI resistance development in PC9 xenograft assay

**Figure 1.**
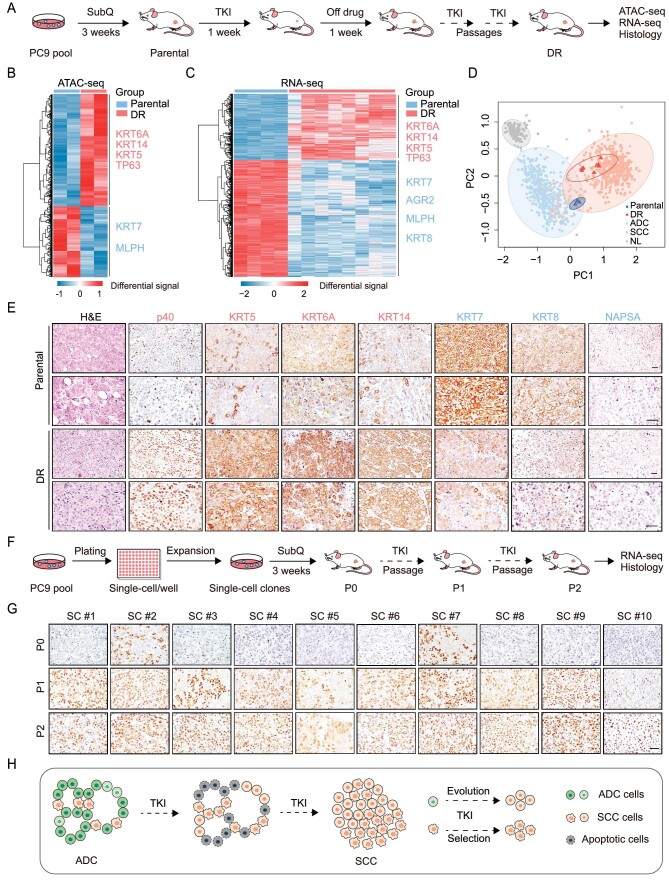
Squamous transition occurs concomitantly with TKI resistance development. (A) Schematic illustration of the establishment and analyses of PC9 xenograft mouse models with EGFR TKI resistance. (B) Heatmap of differential open chromatin regulatory elements (REs) characterized by ATAC-seq in parental (*n* = 2) and DR cells (*n* = 2). Hierarchical clustering yields two clusters of elements and two major groups of samples. The color bar shows the relative ATAC-seq signal (*Z* score of normalized read counts). (C) Heatmap of differentially expressed genes in parental (*n* = 4) and DR tumors (*n* = 8). The color bar shows the relative expression value from RNA-seq data. (D) Principal-component analysis (PCA) of RNA-seq data from parental tumors (*n* = 4), DR tumors (*n* = 8) and human normal lung (NL), ADC and SCC samples from TCGA. (E) Representative H&E and immunostaining in parental and DR tumors. Scale bar, 50 μm. (F) Schematic illustration of the establishment and analyses of single clone-derived PC9 xenograft mouse models with EGFR TKI resistance. (G) Representative p40 immunostaining in single clone-derived PC9 tumors at different passages. (H) A proposed model depicting the AST path in EGFR TKI resistant PC9 xenograft mouse models.

We first established EGFR TKI-resistant models through long-term treatments with gefitinib (GEF) or osimertinib (OSI) in human EGFR-mutant lung cancer cell line PC9 xenograft tumors (Fig. 1A and Fig. S1A and B, F–G). These TKI-resistant tumors exhibited increased levels of Ki-67 (proliferation marker) and decreased levels of cleaved caspase 3 (CC3, apoptosis marker) compared to parental tumors when treated with GEF or OSI (Fig. S1C–E, H–I). We also found that GEF-resistant tumors displayed cross-resistance to OSI (Fig. S1J). We hereafter refer to these EGFR TKI resistant tumors as drug resistant (DR) tumors.

Through assay for transposase-accessible chromatin using sequencing (ATAC-seq), we detected increased chromatin openness of squamous markers (*TP63*, *KRT5*, *KRT6A* and *KRT14*) and reduced chromatin accessibility of adenomatous markers (*KRT7* and *MLPH*) in the DR tumor cells (Fig. 1B and Fig. S1K, M). These findings were further confirmed by RNA-seq analyses and quantitative PCR data (Fig. 1C and Fig. S1L, N). We observed no substantial difference of other squamous markers like *SOX2* and adenomatous markers like *NAPSA* (Fig. S1L, N). In line with previous reports [17], these DR tumors were enriched with squamous markers, and the genes involved in the formation of cell junctions [23] and desmosome [24] (Fig. S2A). Moreover, several other key cancer hallmark signatures, e.g. MYC targets, E2F targets, and MTORC1 signaling were highly enriched in DR tumors (Fig. S2B). Principal component analysis (PCA) of RNA-seq data showed that the DR tumors closely resembled human SCC (TCGA) whereas the parental tumors showed proximity to human ADC (Fig. 1D). These DR tumors displayed high expression of squamous markers (p40 for DNp63, KRT5, KRT6A and KRT14) and low/no expression of adenomatous markers (KRT7, KRT8 and NAPSA) (Fig. 1E and Fig. S2C). We found that EGFR remained phosphorylated in the DR tumors (Fig. S2D). Moreover, we didn't observe other known mechanisms including increased MET expression, epithelial-mesenchymal transition and SCLC transition (Fig. S2E and F). These data indicate that squamous transition concomitantly occurring with long-term TKI treatments in PC9 xenograft assay might contribute to drug resistance.

To explore how squamous transition occurs *in vivo*, we further established single-cell clones from parental PC9 cells, and performed xenograft assays with TKI treatments individually (Fig. 1F and Fig. S3A). Interestingly, we found that 8 out of 10 clones gradually acquired squamous marker expression along with TKI resistance development (Fig. 1G). The other 2 were initially positive for DNp63 and displayed variable degrees of TKI resistance (Fig. 1G). Our RNA-seq data showed that the tumors at passage 2 (P2) displayed upregulation of squamous markers and signature, and closely resembled human SCC (Fig. S3B–E), whereas the initial tumors at passage 0 (P0) were similar to human ADC (Fig. S3C, E). These data, at the single-clone level, further support the capability of squamous transition of PC9-derived xenograft tumors along with TKI resistance development (Fig. 1H).

### Single-cell RNA sequencing data analyses uncover the AST evolution pathway

**Figure 2.**
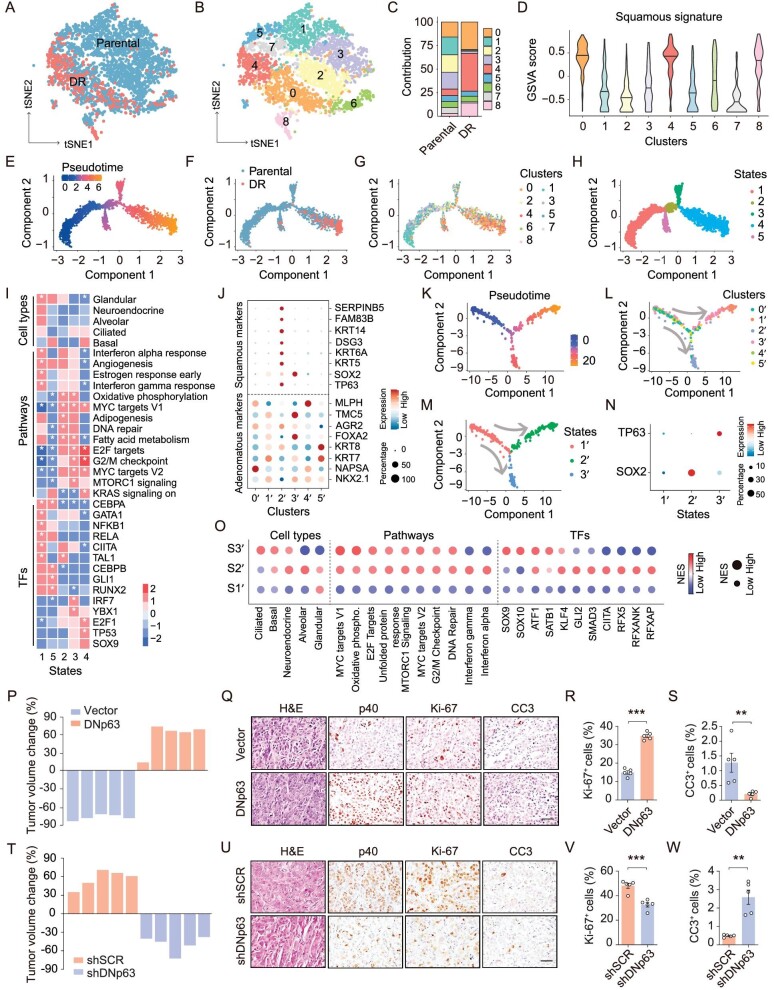
ScRNA-seq delineates the squamous transition process with acquisition of TKI resistance. (A) t-SNE visualization of epithelial cells (parental: *n* = 3416, DR: *n* = 512) from parental and DR tumors scRNA-seq data. (B) t-SNE visualization of nine clusters in parental and DR tumors scRNA-seq data. (C) Clusters distribution in parental and DR tumors scRNA-seq data. (D) Violin plots depicting the squamous signature calculated by GSVA score in each cluster. (E) Pseudotime ordering of tumor cells performed with Monocle2. (F) Pseudotime ordering of epithelial cells in parental and DR tumors with Monocle2. (G) Pseudotime ordering of the nine cell clusters with Monocle 2. (H) Pseudotime ordering of the five states with Monocle 2. (I) Heatmap on enrichment of cell identity, pathways and TFs in the five states with GSEA. The color bar shows the relative normalized enrichment score (NES). Asterisk indicates *P* < 0.05. (J) Violin plots depicting the squamous and adenomatous signature calculated by GSVA score in the six clusters from the Maynard *et al.* scRNA-seq data. (K) Pseudotime ordering of tumor cells performed with Monocle2 from the Maynard *et al.* scRNA-seq data. (L) Pseudotime ordering of the six clusters with Monocle 2 from the Maynard *et al.* scRNA-seq data. (M) Pseudotime ordering of the three states with Monocle 2 from the Maynard *et al.* scRNA-seq data. (N) Dot plot showing expression of TP63 and SOX2 among the three states from the Maynard *et al.* scRNA-seq data. (O) Enrichment of cell identity, pathways and TFs in the three states with GSEA from the Maynard *et al.* scRNA-seq data. Dot color and size shows normalized expression score (NES) in each state compared to others for each pathway. Low represents NES≤−2.5 while high represents NES≥−2.5. (P–S) Tumor volume changes (P), H&E and immunostaining of Ki-67 and CC3 (Q), and related statistical analyses (R–S) in indicated PC9 tumors after 1 week of TKI treatments. Data are shown as mean ± SEM. **P < 0.01; ***P < 0.001; statistical significance was calculated by two-tailed unpaired Student's *t*-test. (T–W) Tumor volume changes (T), H&E and immunostaining of Ki-67 and CC3 (U) and related statistical analysis (V–W) in indicated DR tumors after 1 week of TKI treatments. Data are shown as mean ± SEM. **P < 0.01; ***P < 0.001; statistical significance was calculated by two-tailed unpaired Student's *t*-test.

To further understand the AST process along with TKI resistance development, we performed single-cell RNA sequencing (scRNA-seq) of both parental and DR tumors. Among 9 epithelial clusters identified (Fig. 2A and B), we found that clusters 1, 2 and 3 were mainly enriched in the parental tumors with high adenomatous signature and low squamous signature, whereas clusters 0, 4 and 8 were enriched in the DR tumors with opposite patterns (Fig. 2C and D and Fig. S4A). In contrast, clusters 5, 6 and 7 displayed low levels of adenomatous signature or squamous signature (Fig. 2D and Fig. S4A), indicative of an intermediate state. In line with the pre-existence of TP63 positive cells in single-cell clone-derived tumors (Fig. 1G), scRNA-seq data of parental PC9 tumors also uncovered their existence albeit at a low percentage (2.3%) (Fig. S4L). Consistently, the DR tumors were highly enriched with squamous signature whereas the parental tumors were highly enriched with adenomatous signature (Fig. S4B).

Using monocle trajectory analysis, we further classified these 9 clusters into five distinct states during squamous transition (Fig. 2E–H and Fig. S4C). State 1 showed glandular and alveolar identities, and was enriched with interferon response and metabolic pathways such as fatty acid metabolism (Fig. 2I), consistent with our previous findings of a stressful state during squamous transition [25]. Moreover, we found that state 1 featured high CEBPA activity, which is known to inhibit DNp63 and KRT14 expression, keratinocytes proliferation and the commitment to differentiation [26]. State 2 showed the reduction of glandular signature with a loss of specific cell identity, and the enrichment of the pathways like oxidative phosphorylation and MYC targets, indicative of a transition state [25]. States 3 and 4 were enriched with cell cycle pathways and related TFs including E2F and MYC. Different from state 3 showing ciliated identity, state 4 displayed the basal identity with strong stemness, previously proposed to be the origin of basal-like squamous cell carcinoma [19,27]. State 5 also showed basal identity, TFs (CEBPA, CEBPB and RELA) enrichment, as well as the KRAS signaling enrichment. Unsupervised pseudotime ordering of tumor cells predicted three trajectories: trajectory 1 from state 1 to state 2 to state 3; trajectory 2, from state 1 to state 2 to state 4; trajectory 3, from state 1 to state 5 (Fig. 2H). Although both state 4 and state 5 showed basal identity, only state 4 displayed the significant enrichment of MYC and E2F signaling, indicative of high cell proliferation (Fig. 2H and I). In consideration of the highly proliferative property of squamous tumors, it seemed very likely that trajectory 2 represented the major evolution path of AST. In contrast, trajectory 1 showed an initial loss but later restoration of ciliated epithelia identity (Fig. 2H and I), which is frequently observed during cancer initiation and negatively correlates with cancer malignancy [28–30]. Future detailed investigation is necessary to clarify the biological significance of this trajectory.

We next analyzed the public scRNA-seq data of consecutive biopsies at the treatment naïve (TN), residual disease (RD) and progressive disease (PD) stage from an EGFR-mutant lung cancer patient with squamous transition along with TKI therapy failure (Fig. S4F and G) [31]. In contrast to TN and RD, the tumor biopsy at PD displayed an increase of TP63 expression (Fig. S4D). Among 6 epithelial clusters identified in these tumor samples (Fig. S4H–I), cluster 2′ showed increased expression of squamous markers, and decreased expression of adenomatous markers such as NKX2.1 and NAPSA (Fig. 2J), and this cluster became dominant at the PD stage (Fig. S4J). Trajectory analysis further identified three states along the evolution path (Fig. 2K–M and Fig. S4K). We found that the transition potentially started from state 1′ to state 2′ (S1′–S2′) or state 1′ to state 3′ (S1′–S3′) (Fig. 2M). In the S1′–S3′ route, DNp63 expression gradually increased (Fig. 2M and N), and tumor cells eventually lost glandular identity along with the acquisition of basal identity (Fig. 2O). Pathways analysis revealed that interferon response decreased whereas cell cycle-related pathways including MYC and E2F targets increased. Moreover, we found that immunologic factors CIITA and RFX5 decreased whereas SOX9, SOX10 and ATF1 increased with AST progression (Fig. 2O). These changes in the S1′–S3′ route was consistent with the trajectory 2 (S1–S2–S4) from PC9 tumors. The S1′–S2′ route also showed the acquisition of basal identity and the enrichment of interferon response pathways and TFs including CIITA and RFX5 (Fig. 2M–O). However, the upregulation of SOX2 expression in the S1′–S2′ route was not detectable during the progression from TN or RD to PD stage (Fig. S4E). These data collectively demonstrate that trajectory 2 from PC9 tumors is highly similar to the S1′–S3′ trajectory, which might serve as the major squamous transition path in clinic.

### Perturbation of squamous transition via DNp63 modulates EGFR TKI responsiveness

DNp63 is known as the important squamous lineage specific TF [17,32]. We found that overexpression of DNp63 resulted in increased expression of squamous markers as well as genes involved in tight junction, desmosome and gap junction, along with decreased expression of adenomatous markers and genes involved in extracellular matrix (ECM) receptor interaction in PC9 tumors (Fig. S5A–D). Conversely, knockdown of DNp63 suppressed squamous markers and promoted adenomatous markers (Fig. S6A–C). These results confirmed that the alteration of DNp63 expression can modulate the AST process, consistent with previous findings from genetically engineered mouse models (GEMM) [17].

Importantly, we found that the promotion of the AST process through DNp63 overexpression conferred a strong resistance to TKI treatments on PC9 tumors (Fig. 2P and S5E). In line with this, increased cell proliferation and decreased apoptosis were detectable in DNp63-overexpressing tumors (Fig. 2Q–S). Following TKI treatments, DNp63-overexpressing tumors maintained higher expression of squamous markers and lower expression of adenomatous markers compared with control tumors (Fig. S5F). On the contrary, the AST blockade through DNp63 knockdown re-sensitized the DR tumors to TKI treatments without affecting squamous or adenomatous marker expression (Fig. 2T–W and S6D–E). These findings not only demonstrate the importance of DNp63 in mediating AST, but also reveal a direct causal link between squamous transition and EGFR TKI resistance.

### Identification of the counteracting transcriptional factor network orchestrating the AST process

**Figure 3.**
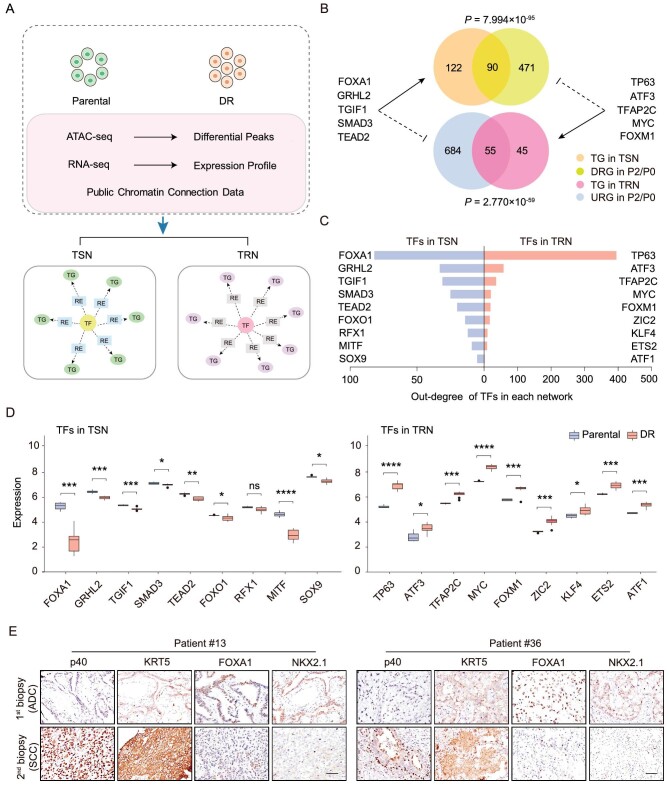
Counteracting transcriptional factor networks orchestrate squamous transition. (A) Schematic illustration of the integrative analyses of RNA-seq and ATAC-seq in parental and DR tumors. TKI sensitive network (TSN) and TKI resistant regulatory network (TRN) were constructed using our previously developed statistical model (see details in Methods). TF, transcription factor; RE, response element; TG, target genes. (B) Mutual counteraction of TSN-enriched and TRN-enriched TF networks based on computation analyses. The significance of overlapped target genes was evaluated with Fisher's exact test. DRG, downregulated genes; URG, upregulated genes; TG, target genes. (C) Enriched TFs in TSN and TRN through integrative analyses of ATAC-seq and RNA-seq data ranked according to the numbers of dysregulated target genes. (D) Gene expression of TFs from TSN and TRN in parental (*n* = 4) and DR tumors (*n* = 8) based on RNA-seq data. Data are shown as mean ± SEM. *P < 0.05; **P < 0.01; ***P < 0.001; ****P < 0.0001; statistical significance was calculated by two-tailed unpaired Student's *t*-test. (E) Representative immunostaining of indicated proteins in two paired human EGFR-mutant lung cancer specimens experiencing squamous transition after EGFR TKI failure (pre- vs. post-transition). Scale bar, 50 μm.

Lineage specific TF networks are proposed to contribute to histologic transition and drug resistance [32–34]. We therefore constructed the dysregulated TF networks through integrative analyses of RNA-seq and ATAC-seq data in the parental and DR tumors [35], and constructed TKI-sensitive TF network (TSN) and TKI-resistant TF network (TRN), respectively (Fig. 3A and Fig. S7A). Interestingly, we found the TSN and TRN networks were mutually counteracting: most genes upregulated by TSN were downregulated by TRN, and *vice versa* (Fig. 3B). RNA-seq data analyses confirmed that the TFs in TSN tended to be lowly expressed in the DR tumors whereas the TFs in TRN showed the opposite pattern (Fig. 3D). Notably, FOXA1 and TP63 were the top-ranked TFs in TSN and TRN, respectively (Fig. 3C). ChIP-seq data analyses identified two FOXA1 binding sites at the *TP63* gene locus (Fig. S7B), in accordance with a previous study [36]. Consistently, FOXA1 knockout significantly upregulated DNp63 transcription in PC9 cells (Fig. S7E). Moreover, protein levels of FOXA1 were downregulated in DNp63-overexpressing tumors but upregulated in DNp63-knockdown tumors (Fig. S7C–D).

We further evaluated the expression of FOXA1 in clinical samples. In the Quintanal-Villalonga *et al.* RNA-seq data [22], we observed decreased FOXA1 expression in post-transition SCC (Fig. S7F). In the Maynard *et al.* scRNA-seq data [31], we also detected reduced FOXA1 expression at the PD stage compared with the TN stage [31] (Fig. S7G). Consistently, both re-biopsy samples with squamous transition [37] also showed reduced FOXA1 expression (Fig. 3E).

### FOXA1 partially inhibits squamous transition and maintains TKI sensitivity

**Figure 4.**
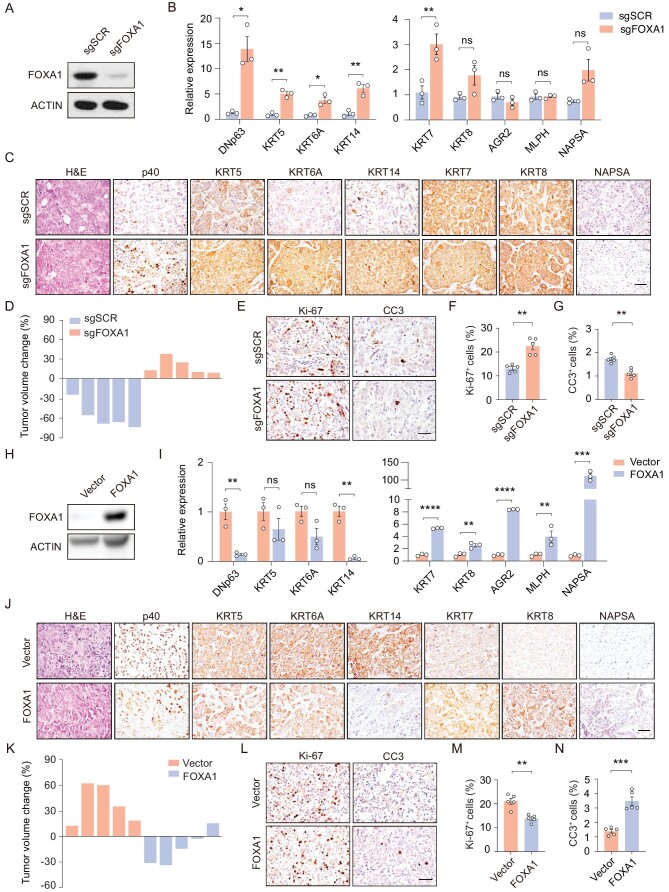
FOXA1 partially inhibits squamous transition and maintains TKI sensitivity. (A) Immunoblotting analysis of FOXA1 in PC9 cells with or without FOXA1 knockout. (B) PCR quantitation of mRNA levels in PC9 control (*n* = 3) and sgFOXA1 tumors (*n* = 3). Data are shown as mean ± SEM. *P < 0.05; **P < 0.01; statistical significance was calculated by two-tailed unpaired Student's *t*-test. (C) Representative H&E and immunostaining in PC9 control and sgFOXA1 tumors. Scale bar, 50 μm. (D) Tumor volume changes in PC9 control and sgFOXA1 tumors after 1 week of TKI (gefitinib) treatments. (E–G) Representative immunostaining staining (E) and statistical analysis of Ki-67 (F) and CC3 (G) in PC9 control and sgFOXA1 tumors after 1 week of TKI (gefitinib) treatments. Data are shown as mean ± SEM. *P < 0.05; statistical significance was calculated by two-tailed unpaired Student's *t*-test. (H) Immunoblotting analysis in DR cells with or without FOXA1 overexpression. (I) PCR quantitation of mRNA levels in DR control and FOXA1-overexpressing tumors. Data are shown as mean ± SEM. **P < 0.01; ***P < 0.001; ****P < 0.0001; statistical significance was calculated by two-tailed unpaired Student's *t*-test. (J) Representative H&E and immunostaining in DR control and FOXA1-overexpressing tumors. Scale bar, 50 μm. (K) Tumor volume changes in DR control and FOXA1-overexpressing tumors after 1 week of TKI (gefitinib) treatments. (L–N) Representative immunostaining (L) and statistical analysis of Ki-67 (M) and CC3 (N) in DR control and FOXA1-overexpressing tumors after 1 week of TKI (gefitinib) treatments. Data are shown as mean ± SEM. *P < 0.05; **P < 0.01; statistical significance was calculated by two-tailed unpaired Student's *t*-test.

We further found that FOXA1 knockout promoted squamous transition (Fig. 4A–C). RNA-seq data showed that FOXA1 knockout led to the upregulation of squamous markers, cell junction- and desmosome-associated signatures (Fig. S8A). Gene set enrichment analysis (GSEA) showed that FOXA1 knockout resulted in the enrichment of cancer hallmark signatures including E2F targets, MYC targets and NOTCH signaling, and other molecular events including cell cycle, DNA replication and keratinization (Fig. S8B–C). Correlation analysis demonstrated a positive correlation between FOXA1-knockout tumors and the DR tumors in both gene expression profiling and chromatin signals (Fig. S8D). Consistently, FOXA1 knockout led to reduced chromatin accessibility at the *AGR2* locus and increased openness at the *TP63* locus (Fig. S8E). In contrast to the parental tumors which exhibited high sensitivity to TKI treatments, the FOXA1-knockout tumors displayed 10%–30% tumor growth and maintained higher expression of squamous markers upon drug treatments, indicating that FOXA1 knockout at least partially conferred TKI resistance (Fig. 4D–G and Fig. S8F–K).

Conversely, we found that FOXA1 overexpression in the DR tumors completely blocked the squamous markers expression while maintaining adenomatous markers expression (Fig. 4H–J). Compared to the DR tumors following TKI treatments, the FOXA1-overexpressing DR tumors displayed higher adenomatous markers expression and lower squamous markers expression, and higher TKI sensitivity (Fig. 4K–N and Fig. S9A–F). These data highlight an important role of FOXA1 in maintaining adenomatous lineage and contributing to TKI sensitivity.

### Concomitant FOXM1 overexpression and FOXA1 knockout fully recapitulates AST and TKI resistance

**Figure 5.**
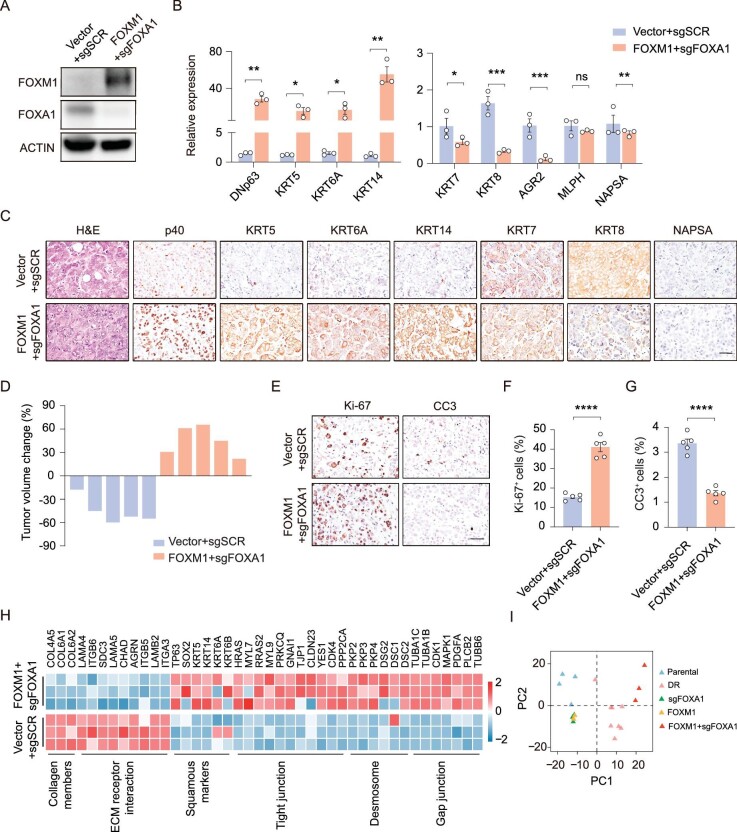
Concomitant FOXM1 overexpression in conjunction with FOXA1 knockout fully recapitulates AST and TKI resistance. (A) Immunoblotting analysis of FOXA1 and FOXM1 in indicated PC9 cells. (B) PCR quantitation of mRNA levels in PC9 control and FOXM1 + sgFOXA1 tumors. Data are shown as mean ± SEM. *P < 0.05; **P < 0.01; ***P < 0.001; statistical significance was calculated by two-tailed unpaired Student's *t*-test. (C) Representative H&E and immunostaining in PC9 control and FOXM1 + sgFOXA1 tumors. Scale bar, 50 μm. (D) Tumor volume changes of PC9 control and FOXM1 + sgFOXA1 tumors after 1 week of TKI (osimertinib) treatments. (E–G) Representative immunostaining (E) and statistical analysis of Ki-67 (F) and CC3 (G) in PC9 control and FOXM1 + sgFOXA1 tumors after 1 week of TKI (osimertinib) treatments. Data are shown as mean ± SEM. ***P < 0.001; statistical significance was calculated by two-tailed unpaired Student's *t*-test. (H) Heatmap of RNA-seq data showing the relative expression of indicated genes in PC9 control and FOXM1 + sgFOXA1 cells. (I) PCA of RNA-seq data from parental tumors (*n* = 4), DR tumors (*n* = 8), and PC9 sgFOXA1 cells (*n* = 3), FOXM1 cells (*n* = 3), and FOXM1 + sgFOXA1 cells (*n* = 3).

We noticed that the squamous transition and resistant phenotype were much weaker in FOXA1-knockout tumors when compared to the DR tumors (Fig. 4D and Fig. S1B), indicative of the involvement of other potential factors. FOX family members, frequently sharing an evolutionarily conserved DNA binding domain [38], are considered to be pioneering factors in cellular reprogramming and have already been implicated in cancer development, progression, metastasis and drug resistance [39,40]. We found that FOXM1, sharing DNA binding motifs with FOXA1 [41,42] (Fig. S10A), was among the top list of the TRN and was also upregulated in the DR tumors (Fig. 3C–D). Although FoxA2 loss, combined with FoxA1 loss, has been reported to drive squamous transition in GEMM [21], it was barely detectable in either parental tumors or the DR tumors (Fig. S10B). Moreover, FOXA1 knockout didn't affect the protein levels of FOXM1 (Fig. S10C). We then focused on the potential role of FOXM1 in AST, and found that FOXM1 overexpression had no major impact upon the protein levels of FOXA1 but did promote the upregulation of squamous markers (Fig. S10D–H) and the tumor volume increased ranging from 10% to 40% upon TKI treatments and maintained higher expression of squamous markers but lower expression of adenomatous markers (Fig. S10I–R). Importantly, FOXM1 overexpression and FOXA1 knockout (FOXM1 + sgFOXA1) led to strong upregulation of squamous markers and downregulation of adenomatous markers (Fig. 5A–C). FOXM1 + sgFOXA1 tumors also showed higher levels of DNp63 expression compared with sgFOXA1 tumors or FOXM1-overexpressing tumors (Fig. S11G–H). When compared with either condition (Fig. 4D and Fig. S10J), combined FOXM1 overexpression and FOXA1 knockout resulted in a notable TKI-resistant phenotype with 30%–80% tumor volume increase and maintained higher expression of squamous markers but lower expression of adenomatous markers upon drug treatments (Fig. 5D–G and Fig. S11A–F), reminiscent of the DR tumors (Fig. S1B). Moreover, we found that the FOXM1 + sgFOXA1 cells and the DR tumors had similar enrichment of squamous signatures (Fig. 5H and Fig. S11I). Cancer hallmark signatures including E2F targets, MYC targets and PI3K-AKT-MTOR, molecular events including cell cycles and DNA replication, were also found highly enriched in FOXM1 + sgFOXA1 cells (Fig. S11J–K). PCA of RNA-seq data further revealed that the FOXM1 + sgFOXA1 cells showed more proximity to the DR tumors (Fig. 5I). These data support that concomitant FOXM1 overexpression and FOXA1 knockout could fully recapitulate the AST and TKI resistance.

**Figure 6.**
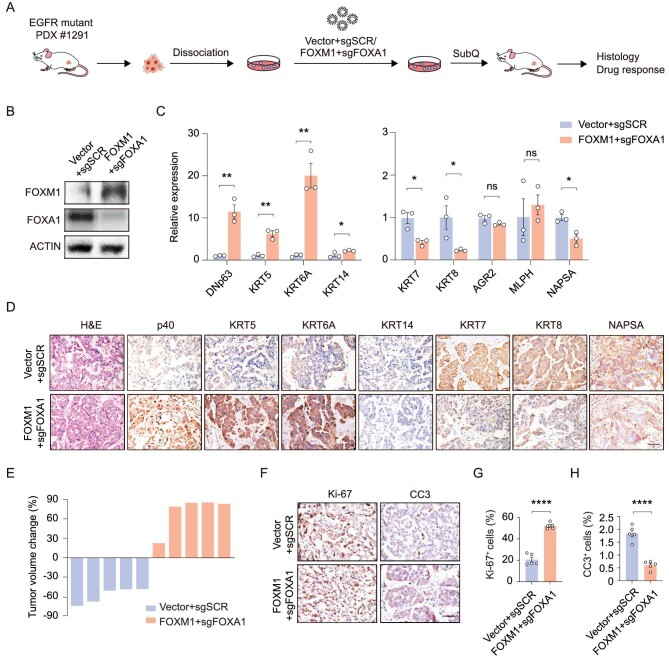
FOXM1 overexpression and FOXA1 knockout confer the EGFR-mutant PDX tumors with TKI resistance. (A) Schematic illustration of lentivirus-mediated transfection of EGFR-mutant PDX tumors. (B) Immunoblotting analysis of FOXA1 and FOXM1 in PDX tumors with FOXM1 overexpression and FOXA1 knockout. (C) PCR quantitation of mRNA levels in control and FOXM1 + sgFOXA1 PDX tumors. Data are shown as mean ± SEM. *P < 0.05; **P < 0.01; statistical significance was calculated by two-tailed unpaired Student's *t*-test. (D) Representative H&E and immunostaining in control and FOXM1 + sgFOXA1 PDX tumors. Scale bar, 50 μm. (E) Tumor volume changes of control and FOXM1 + sgFOXA1 PDX tumors after 1 week of TKI (osimertinib) treatments. (F–H) Representative immunostaining (F) and statistical analysis of Ki-67 (G) and CC3 (H) in control and FOXM1 + sgFOXA1 PDX tumors after 1 week of TKI (osimertinib) treatments. Data are shown as mean ± SEM. ***P < 0.001; ****P < 0.0001; statistical significance was calculated by two-tailed unpaired Student's *t*-test.

To further validate the role of FOXA1 and FOXM1, we established a PDX model (PDX #1291) from EGFR-mutant ADC patients, and performed FOXA1 knockout and FOXM1 overexpression (Fig. 6A and B). We found that the FOXM1 + sgFOXA1 PDX tumor displayed an upregulation of squamous markers and downregulation of adenomatous markers (Fig. 6C and D). Although parental tumor was highly sensitive to TKI treatments, the FOXM1 + sgFOXA1 tumor exhibited strong TKI resistance, with 80% (4/5) tumors showing 60%–90% volume increase upon drug treatments (Fig. 6E–H). Collectively, these findings support that FOXM1 synergizes with FOXA1 loss to promote squamous transition and TKI resistance.

### Identification of RAPGEF3 as a therapeutic target in TKI-resistant tumors

**Figure 7.**
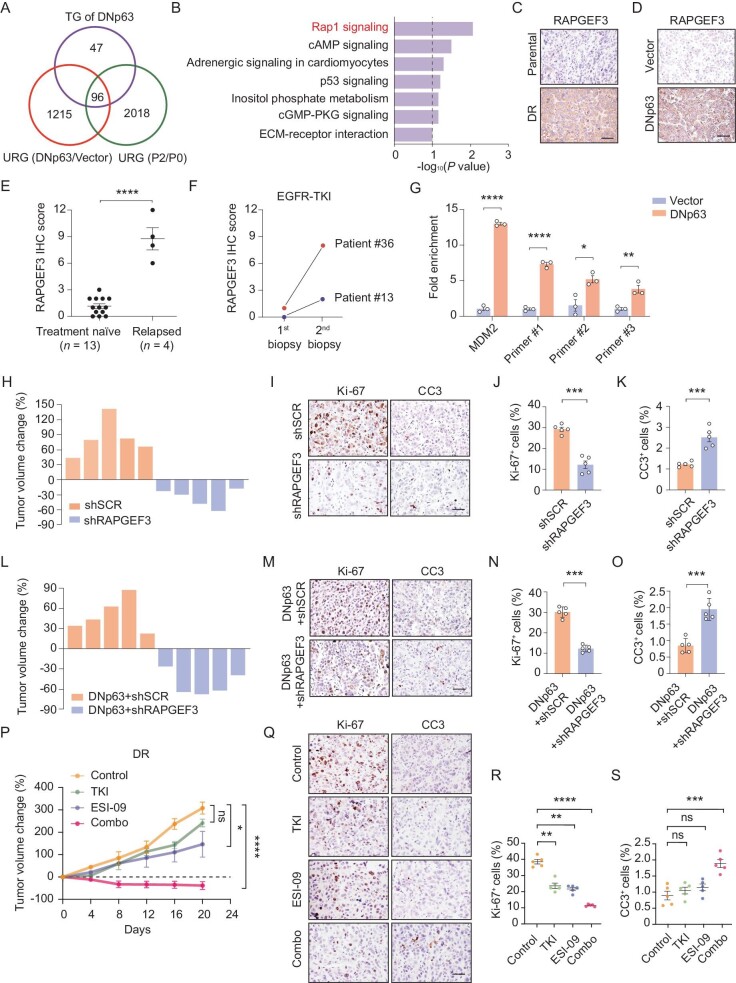
Identification of RAPGEF3 as a therapeutic target in squamous transitioned tumors. (A) Venn diagram showing the integrative analyses of upregulated genes in DNp63-overexpressing cells, single clone derived TKI-resistant tumors (P2/P0) and target genes of DNp63 in TKI resistant networks. TG: target gene. URG: upregulated genes. (B) KEGG pathway analysis was performed using the 96 enriched genes in (A). (C) Immunostaining of RAPGEF3 in parental and DR tumors. (D) Immunostaining of RAPGEF3 in control and DNp63-overexpressing tumors. Scale bar, 50 μm. (E) IHC score of RAPGEF3 in unpaired treatment naïve (*n* = 13) and relapsed (*n* = 4) samples from EGFR-mutant lung cancer patients. (F) IHC score of RAPGEF3 in two paired human EGFR-mutant lung cancer specimens experiencing squamous transition after EGFR TKI failure used in Fig. 3E. First-biopsy: ADC; second-biopsy: SCC. (G) ChIP-qPCR assay for detection of the binding of DNp63 to the promoter region of RAPGEF3 gene in PC9 control and DNp63-overexpressing tumors. (H) Tumor volume changes of DR control and shRAPGEF3 tumors after 1 week of TKI (gefitinib) treatments. (I–K) Representative immunostaining (I) and statistical analysis of Ki-67 (J) and CC3 (K) in DR control and shRAPGEF3 tumors after 1 week of TKI (gefitinib) treatments. Data are shown as mean ± SEM. **P < 0.01; ***P < 0.001; statistical significance was calculated by two-tailed unpaired Student's *t*-test. (L) Tumor volume changes of DNp63-overexpressing tumors with or without RAPGEF3 knockdown after 1 week of TKI (gefitinib) treatments. (M–O) Representative immunostaining (M) and statistical analysis of Ki-67 (N) and CC3 (O) in DNp63-overexpressing tumors with or without RAPGEF3 knockdown after 1 week of TKI (gefitinib) treatments. Data are shown as mean ± SEM. *P < 0.05; **P < 0.01; statistical significance was calculated by two-tailed unpaired Student's *t*-test. (P) Relative tumor growth of DR tumors treated with control, TKI (gefitinib), ESI-09, or combined TKI (gefitinib) + ESI-09. (Q–S) Representative immunostaining (Q) and statistical analysis of Ki-67 (R) and CC3 (S) in DR tumors treated as in (P). Data are shown as mean ± SEM. **P < 0.01; ***P < 0.001; statistical significance was calculated by two-tailed unpaired Student's *t*-test.

To identify potential therapeutic targets to overcome AST-mediated TKI resistance, we performed integrative analyses of upregulated genes (URGs) in DNp63-overexpressing cells, TKI-resistant tumors (P2/P0) and target genes (TGs) of DNp63 in TRN, and identified a significant enrichment of RAP1 signaling (Fig. 7A and B and Table S1). We confirmed the upregulation of the RAP1 signaling-related genes in both the DR and DNp63-overexpressing tumors (Fig. S12A and B). Among the RAP1 signaling-related genes, RAPGEF3, also named EPAC1 (exchange protein directly activated by cAMP1), acts as a guanine nucleotide exchange factor for small GTPases to mediate the intracellular functions of cAMP [43,44] and has been reported to promote proliferation and inhibit apoptosis in multiple cancers [45,46]. Increased openness of the *RAPGEF3* gene locus was observed in DR cells compared to parental cells (Fig. S12C). We also confirmed that RAPGEF3 was upregulated in both DR tumors and DNp63-overexpressing tumors (Fig. 7C and D and Fig. S12D–E) but downregulated in the DNp63-knockdown tumors (Fig. S12F). Additionally, RAPGEF3 was found to be increased in the sgFOXA1, FOXM1 and FOXM1 + sgFOXA1 tumors (Fig. S12G–I) but decreased in FOXA1-overexpressing tumors (Fig. S12J). Analyses of 13 biopsy samples at baseline samples and 4 re-biopsy samples at disease relapse with squamous transition showed that RAPGEF3 expression was significantly higher in the relapsed samples (Fig. 7E). This was further confirmed in two paired pre- and post- squamous transition samples [37] (Fig. 7F and Fig. S12K). Chromatin immunoprecipitation (ChIP) and quantitative PCR analyses further showed that the binding of DNp63 to the *RAPGEF3* promoter region was enriched in DNp63-overexpressing tumors (Fig. 7G and Fig. S12L). These results indicate that RAPGEF3, as a direct downstream target of DNp63, might contribute to AST-mediated drug resistance.

We next performed RAPGEF3 knockdown in the DR cells, and found that RAPGEF3-knockdown tumors were sensitive to TKI treatments (Fig. 7H–K, Fig. S12M and S13A). Moreover, RAPGEF3 knockdown re-sensitized the DNp63-overexpressing tumors to TKI (Fig. 7L–O, Fig. S12N and S13B). We did not observe any notable impact upon adenomatous or squamous markers in the DR tumors or DNp63-overexpressing tumors with RAPGEF3 knockdown (Fig. S13C and D). In addition, we found that RAPGEF3 overexpression in PC9 tumors had no major impact upon squamous and adenomatous markers, as well as TKI responsiveness (Fig. S13E–G). To determine the molecular impact of RAPGEF3 knockdown, we evaluated the EGFR downstream pathways and found that AKT was reactivated in DR tumors and DNp63-overexpressing tumors upon TKI treatments (Fig. S13H and I), and RAPGEF3 knockdown significantly inhibited AKT activation in both DR and DNp63-overexpressing tumors upon TKI treatments (Fig. S13J and K). These data suggest that RAPGEF3 might act as an important downstream effector of DNp63 in mediating EGFR TKI resistance without affecting squamous transition.

### Combined treatments with RAPGEF3 and EGFR inhibitors overcome AST-mediated drug resistance

We next evaluated the therapy targeting RAPGEF3 in TKI-resistant mouse models. ESI-09, a selective inhibitor of RAPGEF3 [47,48], has been reported to possess excellent pharmacological activity [49] and toxicological profile [50,51]. We confirmed the on-target effect of ESI-09 on RAPGEF3 using a thermal shift assay as previously reported (Fig. S14A) [52], and found that ESI-09 treatment alone partially suppressed DR tumors (Fig. 7P). When combined with TKI treatments, ESI-09 resulted in a significant tumor regression without significant weight loss (Fig. 7P and Fig. S14B). Combined treatments also profoundly inhibited AKT activation when compared to monotherapy (Fig. S14C). In line with this, RNA-seq data showed that the combination treatment dramatically downregulated cancer hallmark signatures including MYC targets, E2F targets and MTORC1 signaling (Fig. S14D and E). Remarkable inhibition of proliferation and induction of apoptosis were detectable in the combination treatment group (Fig. 7Q–S).

**Figure 8.**
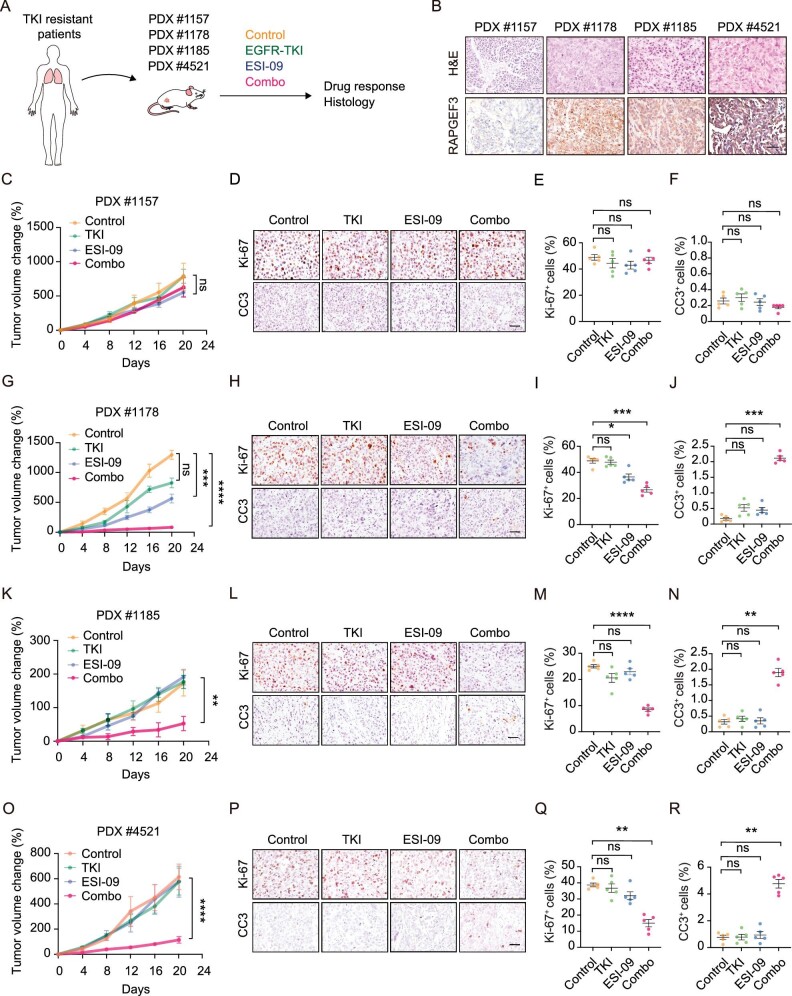
Combined treatments with RAPGEF3 and EGFR inhibitors overcome AST-mediated drug resistance. (A) Schematic illustration of therapeutic strategy in EGFR-TKI-resistant PDXs. (B) Representative H&E and RAPGEF3 immunostaining in EGFR-TKI-resistant PDX tumors. (C–F) Relative tumor growth (C), representative immunostaining (D) and statistical analysis of Ki-67 (E) and CC3 (F) in PDX models (#1157) treated with control, TKI (osimertinib), ESI-09 or combined TKI + ESI-09. (G–J) Relative tumor growth (G), representative immunostaining (H) and statistical analysis of Ki-67 (I) and CC3 (J) in PDX models (#1178) treated with control, TKI (gefitinib), ESI-09 or combined TKI + ESI-09. (K–N) Relative tumor growth (K), representative immunostaining (L) and statistical analysis of Ki-67 (M) and CC3 (N) in PDX models (#1185) treated with control, TKI (osimertinib), ESI-09 or combined TKI + ESI-09. (O–R) Relative tumor growth (O), representative immunostaining (P) and statistical analysis of Ki-67 (Q) and CC3 (R) in PDX models (#4521) treated with control, TKI (osimertinib), ESI-09 or combined TKI + ESI-09. Data are shown as mean ± SEM. *P < 0.05; **P < 0.01; ***P < 0.001; statistical significance was calculated by two-tailed unpaired Student's *t*-test.

We further assessed the efficacy of the combination therapy in multiple TKI-resistant PDX models (Fig. 8A). Among four EGFR-mutant PDX models, three contained acquired TKI resistance (PDX #1157, #1178 and #1185) and one contained intrinsic resistance (PDX #4521) (Fig. 8A). These PDX models displayed variable levels of RAPGEF3 expression (Fig. 8B). We found that the RAPGEF3^high^ PDXs (#1178, #1185 and #4521) showed relatively low expression of adenomatous markers (*NAPSA*, *KRT8* and *AGR2*) and high expression of squamous markers (*DNp63* and *KRT5*) when compared to the RAPGEF3^low^ PDX (#1157) (Fig. S15A). Of note, the combination treatments failed to suppress RAPGEF3^low^ PDX tumors (Fig. 8C–F). In contrast, the combination treatments drastically suppressed RAPGEF3^high^ PDX tumor growth (Fig. 8G, K, O). This was further supported by the proliferation and apoptosis analyses (Fig. 8H–J, L–N, P–R). We found that the combination treatments didn't induce notable body weight loss in mice (Fig. S15B). These data collectively suggest that combined TKI and RAPGEF3 inhibitor treatments is effective in overcoming AST-mediated drug resistance, especially in RAPGEF3^high^ lung cancer.

## DISCUSSION

Multiple studies have implicated a potential link between squamous transition and EGFR TKI resistance [14,15,53]. However, it remains unclear whether AST directly regulates drug resistance. We here find that long term TKI treatments result in drug resistance in PC9 tumors as well as single-cell derived PC9 tumors, and these tumors display the phenotype of concomitant squamous transition. Through integrative RNA-seq, ATAC-seq analyses and functional studies, we find that DNp63 is critical for driving squamous transition, e.g. DNp63 overexpression promotes AST whereas its knockdown has the opposite effect. These findings are in line with the upregulation of DNP63 in EGFR TKI resistant cell lines [54] and supporting the concept that DNp63 acts as a squamous lineage-specific master transcription factor [17,32]. Consistently, we have previously shown that DNp63 overexpression is able to drive AST in Kras/Trp53-mutant GEMM [17]. We find here that the perturbation of squamous transition through DNp63 clearly affects the response to TKI treatments. These data collectively provide direct evidence in supporting the causality between AST and TKI resistance.

Our scRNA-seq data find that clusters 0, 4 and 8 preexisting in parental tumors highly express squamous signatures but are lower in adenocarcinoma signature expression. Moreover, consistent observation of the pre-existence of a few TP63-positive cells in parental tumors as well as single-clone derived tumors demonstrates the PC9 tumor plasticity in xenograft assays. When analyzing human samples with squamous transition after therapy failure, we find that the cluster 2′ enriched with squamous markers is also detectable at the TN stage and becomes dominant at the PD stage. These results indicate that a subpopulation of cells with high plasticity and transition potential might preexist during TKI treatments. In our analysis of clinical samples, the epithelial cells in the PD stage are quite few which may bias the enrichment of cluster 2′ cells in PD. Detailed analyses of a large cohort with AST-mediated drug resistance are warranted in future studies.

Among three trajectory routes identified, we find that trajectory 2 represents a typical evolution route for the AST process: the tumor cells initially experience a transition from alveolar, glandular to basal identity, and then gradually lose stress responsive pathways (the interferon response and fatty acid metabolism pathways, key TFs such as CEBPA, CEBPB, NF-kB and RELA), and eventually reach the high proliferative state (enrichments of MYC and E2F targets). We noticed that two branches form trajectory 1 and 3 along with squamous transition: trajectory 3 acquires basal identity without active cell cycle or related TFs enrichment whereas trajectory 1 regains ciliated identity which is known to be negatively correlated with tumor malignancy. It remains possible that these two branching routes might become unfit due to the cell-cell competition during squamous transition. The importance of trajectory 2 in AST is indeed supported by the analyses of scRNA-Seq data from the patient experiencing AST [31]. We observe an alternative trajectory (S1′–S2′) also gains basal identity with high SOX2 expression. However, we find that SOX2 is not highly expressed at late state, indicating that tumor cells along this trajectory might not have survival advantage and regain sensitivity to TKI treatments.

Our transcriptional network analyses reveal the presence of two distinct TF programs acting in a mutually counteracting manner during the AST process, as exemplified by the roles of FOXA1 and TP63 in regulating AST-related pathways. FOXA1-knockout cells and DNp63-overexpressing cells have shared similar changes in AST-related pathways, including the downregulation of genes involved in ECM receptor interactions and the upregulation of genes involved in tight junction, desmosome and gap junction as well as squamous markers. We identify FOXA1 but not FOXA2 as a dynamically changed TF in TSN in maintaining adenomatous lineage. The downregulation of FOXA1 has been further detected in clinical cohorts [22,31]. A previous study shows that simultaneous knockouts of *Nkx2.1*, *FoxA1* and *FoxA2* could induce squamous transition in *Kras-*driven lung cancer [21]. We notice that both Nkx2.1 and FoxA2 are barely detectable in our system, but FOXA1 knockout could only partially promote AST and TKI resistance. This finding indicates that FOXA1 might need to work in concert with other factors to fully recapitulate this histological transition in EGFR-mutant tumors. FOXM1 has recently been implicated in EGFR TKI resistance [55,56]. We notice that FOXM1 is listed among one of the top TFs in the TKI resistant network maintaining squamous lineage and exhibits an opposite expression pattern to FOXA1. Functional analyses confirm a potential role of FOXM1 in AST: its overexpression leads to the upregulation of squamous markers and drug resistance. More importantly, we find that combining FOXM1 overexpression and FOXA1 knockout fully recapitulates the AST process and TKI resistance. To evaluate how FOXA1 and FOXM1 affects AST or drug resistance, we perform FOXA1 ChIP-seq analysis and detect two FOXA1 binding sites at the *TP63* gene locus. We find that FOXA1 knockout significantly upregulates DNp63 transcription. These findings indicate that FOXA1 might bind to the *TP63* gene locus and transcriptionally repress DNp63 gene expression although future study is necessary to prove this. Moreover, we find that both mRNA and protein levels of DNp63 are higher in FOXM1 + sgFOXA1 tumors than sgFOXA1 tumors or FOXM1-overexpressing tumors. Since DNp63 is critical for inducing AST and TKI resistance, it remains very likely that FOXM1 overexpression together with FOXA1 knockout recapitulates squamous transition and TKI resistance through transcriptional upregulation of DNp63 expression. Future study will be of interest to elucidate detailed mechanisms of how FOXM1 synergizes with FOXA1 inactivation in upregulating DNp63 transcription.

Identification of a targetable molecule in drug-resistant tumors can provide an opportunity to develop novel therapeutic strategies to overcome disease relapse. We demonstrate that RAPGFE3 acts as an important downstream effector of DNp63 in mediating EGFR TKI resistance. RAPGEF3 knockdown recovers the TKI sensitivity of the DR tumors and DNp63-overexpressing tumors without notable impacts upon the AST process. It's worth noting that RAPGEF3 overexpression has no impact upon the expression of squamous and adenomatous markers. Consistently, overexpression of RAPGEF3 alone is insufficient to promote AST or TKI resistance. Nonetheless, RAPGEF3 is necessary for the growth of these tumors with AST and TKI resistance. It remains possible that RAPGEF3 works in concert with other AST-related molecules and/or certain DNp63 downstream regulators in driving AST and AST-related drug resistance. Future efforts are important to dissect these detailed mechanisms. We notice that either EGFR TKI or RAPGEF3 inhibitor alone has marginal inhibitory effect on tumor growth, whereas the antitumor effect is dramatically enhanced upon combinational therapy in RAPGEF3^high^ tumors. Our bioinformatic analyses reveal that oncogenic signaling such as MYC and MTORC1 hallmarks are enriched in DR tumors, and all these pathways are known to be associated with TKI resistance. Combination treatment greatly affects cancer hallmarks such as MYC targets and MTORC1 signaling, and also profoundly inhibits the activation of AKT, which has been implicated in squamous transition of lung adenocarcinoma [22]. RAPGEF3 has been implicated in cancer progression through various downstream signals [57], e.g. RAPGEF3 promotes cancer cell proliferation and/or suppresses apoptosis via Rap1/Akt/CREB signaling [58], Rap1/B-Raf/ERK and mTOR signaling [59] or acting synergistically with PDE4 to promote the Cyclin E1-Cnx43 axis [60]. Alternatively, RAPGEF3 may also function as a pro-inflammatory modulator to promote mTOR-mediated pro-proliferative and anti-apoptotic effects [45]. While our results indicate that combined therapy re-sensitizes drug resistant tumors to EGFR TKI potentially through inhibiting AKT activation, given the complexity of RAPGEF3-mediated signaling cascades, it is possible that multiple mechanisms may act synergistically to contribute to TKI resistance mediated by squamous transition. Further work will be needed to elucidate the detailed mechanism underlying the synergistic effect of combined therapy.

Multiple studies have demonstrated the therapeutic efficacy of ESI-09 in various preclinical animal models including fatal rickettsioses infection [51], chronic inflammatory pain [48] and chemotherapy-induced peripheral neuropathy [49]. Our current study supports the notion that targeting RAPGEF3 could serve as a promising therapeutic strategy for overcoming AST-mediated TKI resistance in EGFR-mutant lung cancer. Emerging evidence has revealed that in addition to EGFR TKI therapy failure, AST has also been observed in other molecular targeted therapy resistance of lung cancer, e.g. in lung cancer patients who have relapsed from ALK inhibitor treatment [61] or Kras^G12C^ inhibitor adagrasib treatment [62]. Given the relatively small sample size of PDX models used in this study, future efforts will be certainly important to validate our findings in a broader range of preclinical AST models and *de novo* lung squamous carcinoma models as well as clinical specimens.

## MATERIALS AND METHODS

### Clinical specimen study

Clinical studies were approved by the Medical Ethics Committee of Shanghai Pulmonary Hospital, Tongji University Medical School Cancer Institute. Written informed consent was obtained from all patients.

Seventeen biopsy specimens including 13 TKI treatment-naïve samples and 4 relapsed samples with squamous transition after TKI failure were used for immunostaining of RAPGEF3 (Fig. 7E). Four biopsy samples from relapsed patients (3 ADC and 1 SCC) with EGFR mutations and one surgical sample from an ADC patient with EGFR mutation were used for PDX establishment and drug treatment study. The PDX #1157 (ADC, Stage IV, EGFR 19del) which was resistant to osimertinib was established from a 58-year-old female. The PDX #1178 (ADC, Stage IV, EGFR 19del) which was resistant to gefitinib was established from a 62-year-old female. The PDX #1185 (ADC, Stage IV, EGFR L858R) which was resistant to osimertinib was established from a 52-year-old female. The PDX #4521 (ADC, Stage IV, EGFR 19del) which was resistant to osimertinib was derived from a 76-year-old male. The surgical sample-derived PDX #1291 (ADC, Stage III, EGFR 19del) showing partial response to osimertinib was established from a 49-year-old male patient and was used for FOXA1 knockout and FOXM1 overexpression functional assay.

Two paired pre- and post-squamous transition biopsy samples were previously reported [37] and used for the immunostaining of p40, KRT5, FOXA1, NKX2.1 and RAPGEF3 (Fig. 3E and Fig. S12K).

### Quantification and statistical analysis

All data represent similar results from three or above independent, biological samples and cell cultures, unless otherwise described. Data were presented as mean ± SEM unless specified. Students *t*-test was used to determine the significance of differences with the annotations. Details of statistical methods for specific analysis are described in the corresponding methods sections.

## Supplementary Material

nwae392_Supplementary_Files

## DATA AND MATERIALS AVAILABILITY

RNA-seq data have been deposited in the OMIX, China National Center for Bioinformation/Beijing Institute of Genomics, Chinese Academy of Sciences (https://ngdc.cncb.ac.cn/omix: accession no. OMIX004548). ATAC-seq data have been deposited under accession no. OMIX004550. Single cell RNA-seq data under accession no. OMIX004549. All data in this paper are available from corresponding author upon reasonable request.
