# Supplementary material for: Integrative study of lung cancer adeno-to-squamous transition in EGFR TKI resistance identifies RAPGEF3 as a therapeutic target
Source: Natl Sci Rev. 2024 Nov 7;11(12):nwae392. doi: 10.1093/nsr/nwae392 (PMC11647589; doi:10.1093/nsr/nwae392)
Supplement: nwae392_Supplementary_Files [file nwae392_supplementary_files.docx]

**Supplementary Data for**

**Integrative study of lung cancer adeno-to-squamous transition in EGFR TKI resistance identifies RAPGEF3 as a therapeutic target**

Hua Wang^1,2,12^, Shijie Tang^1,12^, Qibiao Wu^1,2,12^, Yayi He^3,12^, Weikang Zhu^4^, Xinyun Xie^2,5,6^, Zhen Qin^1^, Xue Wang^1^, Shiyu Zhou^1^, Shun Yao^1^, Xiaoling Xu^7^, Chenchen Guo^1^, Xinyuan Tong^1^, Shuo Han^1^, Yueh-Hung Chou^8^, Yong Wang^4^, Kwok-Kin Wong^9^, Cai-Guang Yang^2,5,6^, Luonan Chen^1,2,10,11,*^, Liang Hu^1,*^ and Hongbin Ji^1,2,10,11,*^

**Supplementary Methods**

**EGFR-mutant human lung cancer cell-derived xenografts and treatment study**

All mice were housed in specific-pathogen-free facilities at the Shanghai Institute of Biochemistry and Cell Biology, and conducted under the guidelines approved by the Institutional Animal Care and Use Committee of the Shanghai Institutes for Biological Sciences, Chinese Academy of Sciences.

For the establishment of xenograft model of EGFR-mutant human lung cancer, PC9 cells (2×10^6^ cells/mice) were inoculated subcutaneously into BALB/c nude mice (BK Co, six-week-old, male). Tumor volumes for subcutaneous tumors were measured with calipers twice per week and calculated using the formula: (width)^2^× length/2. For the assessment of relative tumor volume changes, we defined tumor volume pre-treatment as 1 (100%), and relative tumor volume change of pre- and post- TKI treatments were calculated using the following formula:

Tumor volume change (%) =$\frac{Vpost-Vpre}{\mathrm{Vpre}}\times100\%.$

For the establishment of the DR tumors, gefitinib (25 mg/kg) or osimertinib (5 mg/kg) or vehicle (1% Tween-80) were given orally to mice when the PC9 tumor volume reached 100-200mm^3^. In detail, PC9 xenograft bearing mice were treated with gefitinib or osimertinib for one week and followed by one week off-drug, which is considered as a cycle. Multiple cycles of treatments were given to mice until no obvious tumor regression in response to drug was observed.

PC9 single cells were picked up and grown until ready for the single cell clone-derived xenograft assay. A total of 10 single-cell derived clones (2×10^6^ cells/mouse) were injected into the right flank of nude mice respectively. Drug was given orally until no obvious tumor shrinkage as above.

For the gene modulation study, PC9 cells (2×10^6^ cells/mouse) transfected with pCDH-DNp63, pCRISPRV2-sgFOXA1, pCDH-FOXM1, or pCDH-FOXM1+pCRISPRV2-sgFOXA1 respectively, or the DR cells (2×10^6^ cells/mouse) transfected with pCDH-shDNp63 or pCDH-FOXA1 were mixed with matrigel (1:1) and subcutaneously injected into the right flank of nude mice for xenograft assays to assess the effects of modulation of these factors upon squamous transition and EGFR TKI response respectively. When tumor volume reached 100-200 mm^3^, mice were sacrificed and tumors were dissected for molecular and pathological analyses. Mice were also given gefitinib (25 mg/kg) or osimertinib (5 mg/kg) orally for indicated time and analyzed pathologically.

For the establishment of EGFR-TKI resistant PDX models, human biopsies samples were collected, embedded in matrigel and transplanted into the flanks of NOD-SCID mice (SLAC Co, six-week-old, female).

For the establishment of FOXM1+sgFOXA1 PDX, the PDX #1291 was digested in collagenase digestion buffer and the resultant cells were passed through 70 μm and 40 μm cell strainers and centrifuged, followed by incubated in RBC lysis buffer and resuspended in medium. Cells were then transfected with FOXM1 and sgFOXA1 lentivirus for 48 hours and mixed with Matrigel (1:1) and transplanted into the flanks of NOD-SCID (SLAC Co, six-week-old, female) mice for xenograft assays to assess the effects of FOXM1+ sgFOXA1 upon squamous transition and TKI response.

For combination treatments, mice were treated with vehicle (1% Tween-80), ESI-09 (10 mg/kg), gefitinib (25 mg/kg, for DR tumor and PDX #1178) or osimertinib (5 mg/kg, for PDX #1157, #1185 and #4521), or ESI-09 (10 mg/kg) +gefitinib (25 mg/kg) or ESI-09 (10 mg/kg) +osimertinib (5 mg/kg) orally, daily for 3 weeks. Tumors were collected for molecular and pathological analyses.

**Cell culture**

PC9 cells were purchased from ATCC and were free of mycoplasma contamination. PC9 drug-sensitive (parental) and TKI-resistant (DR) cells were generated as follows: freshly isolated parental and DR tumors were minced with sterile razor blades and cultured in DMEM with 10% fetal bovine serum (FBS) and 1% Penicillin/Streptomycin. All studies were done on cells maintained in culture for less than ten passages. Cell lines were grown in DMEM (Hyclone) with 8% FBS (GIBCO).

For gene overexpression assay, human DNp63, FOXA1, FOXM1 and RAPGEF3 cDNAs were amplified through PCR using the cDNA from normal human cell line 293T cells as the template. The primers used for these PCR were described in Table S2. All cDNAs were cloned into the lentiviral vector PCDH-CMV-neomycin (Addgene) for transfection. After 48h indicated lentivirus transfection, cells were treated with G418 (500 μg/ml, Sigma) for 5 days for selection of transfected cells.

For gene knockdown assay, shRNA constructs from Gene-ray Company were used and the sequences were described in Table S2. All shRNAs were subcloned into the pCDH-U6-puromycin vector for transfection. After 48h lentivirus transfection, cells were treated with puromycin (3 μg/ml, Sigma) for 2 days for selection of transfected cells.

For gene knockout assay, sgRNA constructs from Gene-ray Company were used and sequences were described in Table S2. All sgRNAs were subcloned into the pCRISPRv2 lentiviral vector for transfection. After 48h lentivirus transfection, cells were treated with puromycin (3 μg/ml, Sigma) for 2 days for selection of transfected cells.

Empty pCDH, pLKO.1 and/or pCRISPRv2 vector were used as controls. Lentiviral delivery of ectopic expression plasmid, shRNAs and sgRNAs directed against genes were performed as described previously [1]. Plasmids were packaged into lentiviral particles by co-transfection with packaging plasmids into HEK293T cells and the filtered cell culture supernatant was then used to infect cells.

**Bulk RNA-seq sample preparation and data analysis**

Parental tumor, DR tumor, single clone derived P0 and P2 tumor samples were freshly dissected and preparation for RNA extraction using TRIzol reagent. Cell samples including control, DNp63-overexpressing cells, sgFOXA1, FOXM1, FOXM1+ sgFOXA1 were collected using TRIzol reagent. The RNA-seq libraries and sequencing were constructed according to the standard Illumina RNA-seq protocol (NovaSeq 6000, Berry Genomics, Inc.).

Raw RNA-seq data were aligned to hg38 human genome reference using STAR (v2.6.0) [2]. Depth correction and log2 normalization were performed on raw count data. Genes expressed as zero in more than 85% samples were filtered out. Potential batch effects were corrected with Combat function in SVA package [3]. Differential pathways analysis under different conditions was performed by limma [4] on Gene set variation analysis (GSVA) enrichment scores calculated by R package GSVA [5](limma powers differential expression analyses for RNA-seq and microarray studies).

Public RNA-seq datasets (https://drive.google.com/drive/folders/1YX6jNesWy_rsdx4

6eErkc_bHEymqNVia?usp=sharing) of human pre- and post-squamous transition samples were derived from Quintanal-Villalonga et al. study [6] and used for the analyses for FOXA1 expression. Raw fastq data were aligned to hg38 human genome reference using STAR (v2.6.0) [2] and then count data was processed with depth correction and log2 normalization.

**ATAC-seq sample preparation and data analysis**

Parental cells, DR cells, control and sgFOXA1 cells (80,000 cells) were freshly collected and lysed for 3 minutes using 0.1% NP40 in resuspension buffer and then spun down at 500g for 10 minutes at 4°C. Tn5 transposition of nuclei pellets was carried out at 37°C for 30 minutes and then library was constructed using TruePrep DNA library preparation Kit V2 for Illumina (Vazyme). Sequencing was performed according to the standard Illumina RNA-seq protocol (NovaSeq 6000, Berry Genomics, Inc.).

Raw ATAC-Seq pair-end reads were trimmed for Illumina adaptor sequences and transposase sequences using a customized script and mapped to hg19 using bowtie [7] with parameters -S -X2000 -ml. Duplicate reads were discarded with samtools rmdup [8]. Peaks were identified using MACS2 with -f bed -q 0.01 -nomodel-shift 0. Differentially accessible peaks from the merged union peak list were identified with the edgeR package (Bioconductor) using raw counts of each sample in the union peak list.

**ScRNA-seq data analysis**

Parental and DR tumors were freshly collected, cut and then minced in 5mL lysis buffer (5 mg/mL Collagenase Type II; 1 mg/mL DNase I; 10 μM Y27632) for 1 hour in gentleMACS Dissociator (Miltenyl Biotec). The dissociated cell suspension was filtered through 70 μm and 40 μm filters and spun down at 1200 rpm for 5 minutes at 4°C. Cell pellets were then resuspended and lysed in Red Blood Cell lysis buffer (Beyotime) for 5 minutes and terminated with 10% FBS medium and spun down at 1200 rpm for 5 minutes. After washing cell pellets with FACS buffer (2% FBS; 1xPBS; 10 μM Y27632), the pellet was resuspended in 50 μL FACS buffer and stained with EpCAM (Invitrogen, #2285411) for 30 minutes at 4°C. EpCAM^+^ tumor cells were sorted using FACSAria Fusion (BD). Cells were counted with Countess™ II Automated Cell Counter (Thermo Fisher) and evaluated for viability. The scRNA-seq libraries and sequencing were constructed according to the standard Illumina RNA-seq protocol (NovaSeq 6000, Berry Genomics, Inc.).

Raw FASTQ profiles was processed using Cell Ranger software (v6.0.2) to align to GRCh38 reference genome and count unique molecular identifiers (UMI). Low-quality cells were filtered (expressing fewer than 200 genes or more than 5000 genes, < 1000 gene counts and >20% mitochondrial reads). As a result, 36601 genes in a total of 4438 cells were detected in sample Parental and 36601 genes in a total of 982 cells were detected in sample DR. Epithelial cells from these two samples were then merged and normalized with Seurat package (v4.0.4). Monocle trajectory analysis was performed with Monocle 2 based on differentially expressed genes between these two samples [9]. Enrichment score of squamous signature and adenocarcinoma signature in each cell were calculated with Gene set variation analysis (GSVA) [5]. Squamous signature genes (SOX2, TP63, KRT6A, KRT14, KRT5, DSG3, FAM83B, CLCA2, SERPINB5) and adenocarcinoma signature genes (NKX2.1, FOXA1, NAPSA, KRT8, KRT7, FOXA2, AGR2, TMC5, MLPH) were defined as previous researches [10,11]. Gene set enrichment analysis (GSEA) was performed with R package clusterProfiler to determine cancer hallmark signatures (MSigDB) and transcription factors (TRRUST) specifically enriched in each cluster with ranked genes list (one compared with others). ClusterProfiler 4.0: a universal enrichment tool for interpreting omics data; Gene set enrichment analysis: a knowledge-based approach for interpreting genome-wide expression profiles; The Molecular Signatures Database (MSigDB) hallmark gene set collection; TRRUST: a reference database of human transcriptional regulatory interactions.

Public scRNA-seq datasets (Github: czbiohub/scell_lung_adenocarcinoma) of TN, RD and PD samples from an EGFR-mutant lung cancer patient with squamous transition along TKI therapy failure were derived from Maynard et al. study and used for the analyses for clusters distribution, pseudotime ordering, cell identity and pathway enrichment and TFs expression. Briefly, raw expression profile of public single-cell RNA sequencing data on an EGFR-mutant lung cancer patient was downloaded and filtered according to Maynard et al. (remaining cells with nGenes >= 500 and nReads >= 50000) [12]. As a result, 26485 genes in a total of 2576 cells were detected and 592 cells were recognized as epithelial cells and remained in subsequent analysis (TN=21, RD=555, PD=16). GSEA was performed with R package clusterProfiler to determine cancer hallmark signatures (MSigDB) and transcription factors (TRRUST) specifically enriched in each cluster. Monocle trajectory analysis was performed with Monocle 2 based on differentially expressed genes in RD samples.

**ChIP-seq library preparation and data analysis**

Parental PC9 cells and DR cells (1×10^7^ cells) were fixed with 1% formaldehyde at room temperature for 10 minutes with rotation, and 125 mM glycine was then added to quench the formaldehyde at room temperature for 5 minutes. A volume of 130 μl ice-cold lysis buffer (1% SDS, 10 mM EDTA, 50 mM Tris-HCl, 1× proteinase inhibitor) was added and the content was then transferred into a Covaris milliTUBE 130 μl AFA Fiber vial and incubated at 4°C for 1 hour. Cells were sheared in a Covaris S220 ultra-sonicator (target BP: 300, PIP: 140, duty falter: 10%, cycles/BURST: 200, time: 120s). Clarified samples were collected by centrifugation at 12,000 rpm for 15 minutes at 4°C. After preclearing with 30 μL of protein G beads (Invitrogen, 10003D), 3 μg of anti-FOXA1 (Abcam, ab23738) antibody was added for immunoprecipitation overnight. To bind the anti-FOXA1 antibody, 100 μL of protein G beads was added and incubated with rotation for 3 hours at 4°C. The beads were washed twice each with Low Salt Wash Buffer, High Salt Wash Buffer and LiCl Wash Buffer and resuspended in 100 μL of freshly prepared DNA Elution Buffer (50 mM NaHCO3 and 1% SDS). The ChIP sample beads were placed on a magnet, and the supernatant was collected into a new tube. The above elution step was repeated with another 100 μL of elution buffer. The samples were then digested with 1 μL of Proteinase K (Invitrogen, 25530049) with incubation at 65°C for 5 hours. DNA was purified with Hipure Gel Pure DNA Mini Kit (Magen, D2111-03) and library construction was performed with a TruePrep DNA Library Prep Kit V2 for Illumina (Vazyme, TD503). The libraries were sequenced with the Illumina NovaSeq sequencing system (PE 2×150 bp reads) at Berry Genomics.

Raw chip-seq data were processed with Trimmomatic (v0.39) [13] for adapter trimming and low quality reads filtering, and then mapped to the human reference genome hg19 with Bowtie2 (v2.3.1) [14]. Duplicate reads are removed with sambamba (v0.6.6) [15]. Peak calling was conducted with MACS2 (v2.1.1) and the threshold was set as q<0.05 [16]. DeepTools bam Coverage (with parameters –normalize using RPKM) was used to converted bam files into bigwig format and then deep tools plot heatmap was used for visualization [17].

**TF-RE-TG regulatory Network construction**

We used a previously developed statistical model of TF-RE-TG regulatory networks [18,19] to integrate RNA-seq, ATAC-seq to infer the difference between two populations (parental versus DR). This model quantifies the process that each RE interacts with relevant TFs to affect the expression of its TG. We started with a differentially accessible peak list called from ATAC-seq across all samples as our REs in the following analysis. Next, we identified the upstream TFs and downstream genes for a RE, treated each TF-RE-TG triplet as the basic regulatory unit, ranked them by integrating genomic features, and extracted the significant regulatory relations. Our model is based on several assumptions. RE openness was defined as the fold enrichment of the read starts in this region versus the read starts in a 1M bp background window. Each TF was described by its motif binding score to the RE and its expression level from the FPKM value of RNA-seq experiments. The regulation of a TF on a RE was associated by the motif occurrence in the RE and quantified by the motif binding affinity.

Step 1: Finding TF-RE-TG triplet

For TF-RE pairs, we use HOMER to scan differentially accessible regions to find all positions with substantial similarity to TF’s sequence motif or position weight matrix (PWM). Then we assembly TF-RE pairs with binding strength evaluating its significance. For RE-TG pairs, we consider both proximal regulation and distal regulation. In each population, we use HOMER to annotate differential peaks with nearest genes and select peaks within 5 Kb of TSS to construct the proximal RE-TG regulation pair. Thus, through matching TF-RE pair and RE-TG pair, we can find all candidate TF-RE-TG triplet in network.

Step 2: Collecting genomic features from ATAC-seq and RNA-seq data

After finding all TF-RE-TG triplet, we define a regulatory score and rank triplets in order to obtain significant triplet involve in differential regulation among populations. From RNA-seq data and ATAC-seq data, we collected various genomic features, including TF expression change, TG expression change, openness changes of REs, TF bindings derived from motif occurrence.

Step 3: Integrate genomic features and rank TF-RE-TG triples

Our aim is to model how a TF will regulate a TG via REs with conditions measurement in matched ATAC-seq and RNA-seq data. Given one TF-RE-TG triplet, we assumed that TF may regulate this TG’s expression by REs. Thus, we can collect the genomic features for TF-RE-TG triplet in drug-sensitive and drug-resistant conditions and calculate the fold change for openness of RE and expression of TF and TG.

With those features, we assumed normal distribution for each feature across all the triplets and those transformed features are independent. Using Fisher’s method, we can combine the features into score S.

S=$\sum_{i=1}^{4} Pi$

Where P_i_ is the -log(p-value) for the i-th hypothesis test to assess the significance level of feature i. When the p-value tend to be small, the test statistic S will be large, which suggests that TF-RE-TG regulation is significant. S follows a chi-squared distribution with 2K degrees of freedom, from which a p value for the global hypothesis can be easily obtained, K is the number of features being combined (K=4 in our case). As a result, all the triplets can be ranked by score S and convert score S into p value.

Step 4: Extracting significant TF-RE-TG triplets into network for visualization

By taking a cutoff p value<0.05, we predicted a set of TF-RE-TG triplets. Pooling all the triplets together, we then have a TF-RE-TG network, where TF and TG are nodes, and RE and RE is the edge. We respectively do statistics on the nodes number of TF and rank in each population. Besides, the TF-TG networks are visualized by Cytoscape [20] in order to make the results more clearly.

**Quantitative PCR analysis**

Total RNAs were extracted from cells using TRIzol reagent (Life Technologies), and retro-transcribed into first-strand cDNA using the first-strand synthesis system following the manufacturer’s protocol (Invitrogen, Carlsbad, CA). Quantitative real-time PCR was performed using Roche LightCycle 96 with SYBR Green Master Mix (Roche) following the manufacturer’s manual. The sequences of primers were listed in Table S2. The expression of each gene was normalized to GAPDH. Ectopic expression and knockdown efficiency were also analyzed by quantitative PCR through normalization to the control group. Experiments were done in quadruplicate. Data are presented as mean ± SEM.

**Western blotting assays**

Protein extracts (40 μg) obtained from tumor tissue or cell lines were separated on SDS/PAGE gels (Thermo Fisher Scientific), transferred to a nitrocellulose membrane and blotted with antibodies raised against DNp63 (Maxim, RMA-0815), FOXA1 (Abcam, ab170933), FOXM1 (Abcam, ab207298), EPAC1 (Cell Signaling Technologies, 28366), β-actin (Abcam, AB136452). Primary antibodies were detected against mouse or rabbit IgGs (HRP, Dako and Alexa Fluor 680, Invitrogen) and visualized with ECL Western blot detection solution (GE Healthcare).

**Immunohistochemical staining**

For routine histological analysis, specimens were fixed in 4% buffered formalin (Sigma) and embedded in paraffin. Tissues were serially sectioned (5 μm thick) and stained by conventional H&E every ten sections. Antibodies used for immunostaining included those raised against p40 (Maxim, RMA-0815), KRT5 (Bioworld, BS1208), KRT6A (BBI Life Science, D220238), KRT14 (Covance, PRB-155P), KRT7 (Abcam, ab181598), KRT8 (Abcam, ab53280), Napsin A (Maxim, MAB-0704), NKX2.1 (Abcam, ab133638), NCAM (Abclone, A0393), pEGFR Y1086 (Bioworld, BS4796), FOXA1 (Abcam, ab170933), FOXM1 (Abcam, ab207298), Ki67 (Leica Biosystems, NCL-Ki67p), Cleaved Caspase3 (Cell Signaling Technologies, 9661), RAPGEF3 (Cell Signaling Technologies, 28366). For analysis RAPGEF3 expression, immunostaining was blindly scored using German immunoreactive score as described previously [21]. Briefly, the staining intensity was scored in four categories: “0” (no staining), “1” (weak positive staining), “2” (intermediate positive staining) and “3” (strong positive staining) and the staining extent was graded as “0” (<5%), “1” (5%-25%), “2” (25%-50%), “3” (50%-75%) or “4” (>75%). The scores of the staining intensity and the staining extent were multiplied to give a final IHC score of 0-12. Cases with discrepancies in IRS score were discussed together with other pathologists until consensus was reached.

**Chromatin immunoprecipitation Assay**

Cells were cross-linked with 1% formaldehyde for 5 minutes at room temperature, lysed by SDS lysis buffer and sonicated to generate DNA fragments with an average size of 200-500 bp. After pre-clearing with protein A/G beads, antibody against p63 (Proteintech, 12143-AP), rabbit IgG (CST-3900, Cell Signaling Technologies) was added to cell lysate and incubated at 4°C overnight. DNA cross-linked with antibodies was then pulled down with Protein A/G beads, washed, and purified with MinElute PCR purification kit (NO.28004, QIANGEN). Aliquots of ChIP-enriched DNA and whole-cell lysate DNA were subjected to quantitative PCR analyses.

**Supplementary References**

1. Gao Y, Zhang W, Han X *et al.* YAP inhibits squamous transdifferentiation of Lkb1-deficient lung adenocarcinoma through ZEB2-dependent DNp63 repression. *Nat Commun* 2014; **5**:4629.

2. Dobin A, Davis CA, Schlesinger F *et al.* STAR: ultrafast universal RNA-seq aligner. *Bioinformatics* 2013; **29**:15–21.

3. Leek JT, Johnson WE, Parker HS *et al.* The sva package for removing batch effects and other unwanted variation in high-throughput experiments. *Bioinformatics* 2012; **28**:882–3.

4. Ritchie ME, Phipson B, Wu D *et al.* limma powers differential expression analyses for RNA-sequencing and microarray studies. *Nucleic Acids Res* 2015; **43**: e47.

5. Hänzelmann S, Castelo R, Guinney J. GSVA: gene set variation analysis for microarray and RNA-seq data. *BMC Bioinformatics* 2013; **14**:7.

6. Quintanal-Villalonga A, Taniguchi H, Zhan YA *et al.* Comprehensive molecular characterization of lung tumors implicates AKT and MYC signaling in adenocarcinoma to squamous cell transdifferentiation. *J Hematol Oncol* 2021; **14**:170.

7. Langmead B, Salzberg SL. Fast gapped-read alignment with Bowtie 2. *Nat Methods* 2012; **9**:357–9.

8. Li H, Handsaker B, Wysoker A *et al.* The Sequence Alignment/Map format and SAMtools. *Bioinformatics* 2009; **25**:2078–9.

9. Trapnell C, Cacchiarelli D, Grimsby J *et al.* The dynamics and regulators of cell fate decisions are revealed by pseudotemporal ordering of single cells. *Nat Biotechnol* 2014; **32**:381–6.

10. Tang S, Xue Y, Qin Z *et al.* Counteracting lineage-specific transcription factor network finely tunes lung adeno-to-squamous transdifferentiation through remodeling tumor immune microenvironment. *Natl Sci Rev* 2023;**10**: nwad028.

11. Pan Y, Han H, Hu H *et al.* KMT2D deficiency drives lung squamous cell carcinoma and hypersensitivity to RTK-RAS inhibition. *Cancer Cell* 2023; **41**:88-105.e8.

12. Maynard A, McCoach CE, Rotow JK *et al.* Therapy-Induced Evolution of Human Lung Cancer Revealed by Single-Cell RNA Sequencing. *Cell* 2020; **182**:1232-1251.e22.

13. Bolger AM, Lohse M, Usadel B. Trimmomatic: a flexible trimmer for Illumina sequence data. *Bioinformatics* 2014; **30**:2114–20.

14. Langmead B, Trapnell C, Pop M *et al.* Ultrafast and memory-efficient alignment of short DNA sequences to the human genome. *Genome Biol* 2009; **10**: R25.

15. Tarasov A, Vilella AJ, Cuppen E *et al.* Sambamba: fast processing of NGS alignment formats. *Bioinformatics* 2015; **31**:2032–4.

16. Zhang Y, Liu T, Meyer CA *et al.* Model-based analysis of ChIP-Seq (MACS). *Genome Biol* 2008; **9**: R137.

17. Ramírez F, Dündar F, Diehl S *et al.* deepTools: a flexible platform for exploring deep-sequencing data. *Nucleic Acids Res* 2014; **42**: W187-191.

18. Jin Y, Zhao Q, Zhu W *et al.* Identification of TAZ as the essential molecular switch in orchestrating SCLC phenotypic transition and metastasis. *Natl Sci Rev* 2022;**9**: nwab232.

19. Li L, Wang Y, Torkelson JL *et al.* TFAP2C- and p63-Dependent Networks Sequentially Rearrange Chromatin Landscapes to Drive Human Epidermal Lineage Commitment. *Cell Stem Cell* 2019; **24**:271-284.e8.

20. Shannon P, Markiel A, Ozier O *et al.* Cytoscape: a software environment for integrated models of biomolecular interaction networks. *Genome Res* 2003; **13**:2498–504.

21. Tang L, Tan Y-X, Jiang B-G *et al.* The prognostic significance and therapeutic potential of hedgehog signaling in intrahepatic cholangiocellular carcinoma. *Clin Cancer Res* 2013; **19**:2014–24.

**Supplementary Figures and Legends**


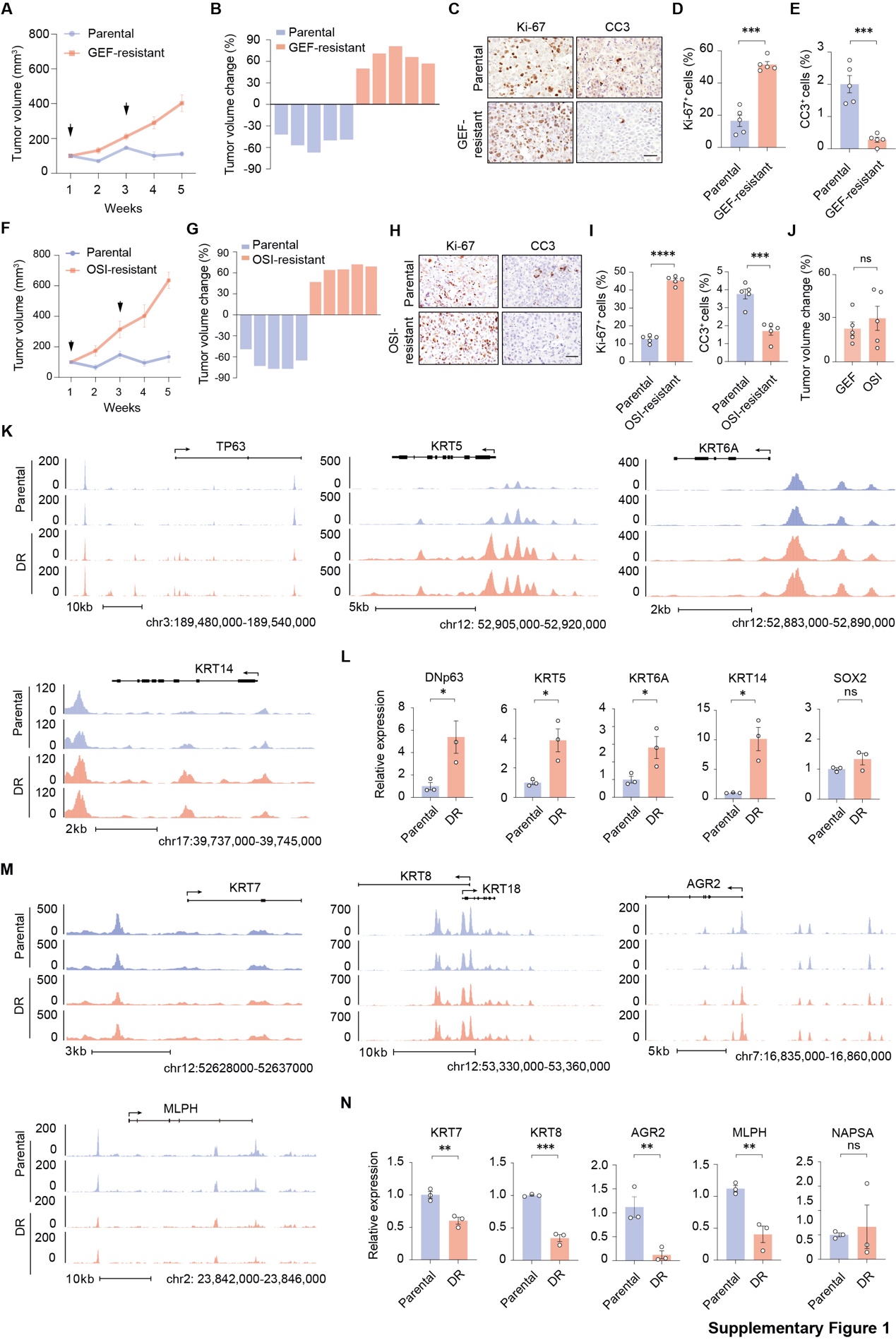


**Supplementary Figure 1. Squamous transition occurs concomitantly with TKI resistance development in PC9 pool xenografts**

(**A**) Relative tumor growth of parental tumors and gefitinib (GEF)-resistant tumors treated with gefitinib. (**B**) Tumor volume changes of parental tumors and GEF-resistant tumors after 1 week of gefitinib treatment. (**C**, **D** and **E**) Representative immunostaining (**C**) and statistical analysis for Ki-67 (**D**) and CC3 (**E**) in indicated tumors. (**F**) Relative tumor growth of parental tumors and osimertinib (OSI)-resistant tumors treated with osimertinib. (**G**) Tumor volume changes of parental tumors and OSI-resistant tumors after 1 week of osimertinib treatment. (**H** and **I**) Representative immunostaining (**H**) and statistical analysis for Ki-67 (**I**, left) and CC3 (**I**, right) in indicated tumors. (**J**) Relative tumor volume change of GEF-resistant tumors treated with gefitinib or osimertinib. (**K**) Normalized ATAC-seq profiles in parental (n=2) and DR (n=2) cells. (**L**) PCR quantitation of mRNA levels in parental (n = 3) and DR (n = 3) tumors. Data are shown as mean ± SEM. *p < 0.05; Statistical significance was calculated by two-tailed unpaired Student’s *t*-test. (**M**) Normalized ATAC-seq profiles in parental (n=2) and DR (n=2) cells. (**N**) PCR quantitation of mRNA levels in parental tumors (n = 3) and DR tumors (n = 3). Data are shown as mean ± SEM. **p < 0.01; ***p < 0.001; Statistical significance was calculated by two-tailed unpaired Student’s *t*-test.


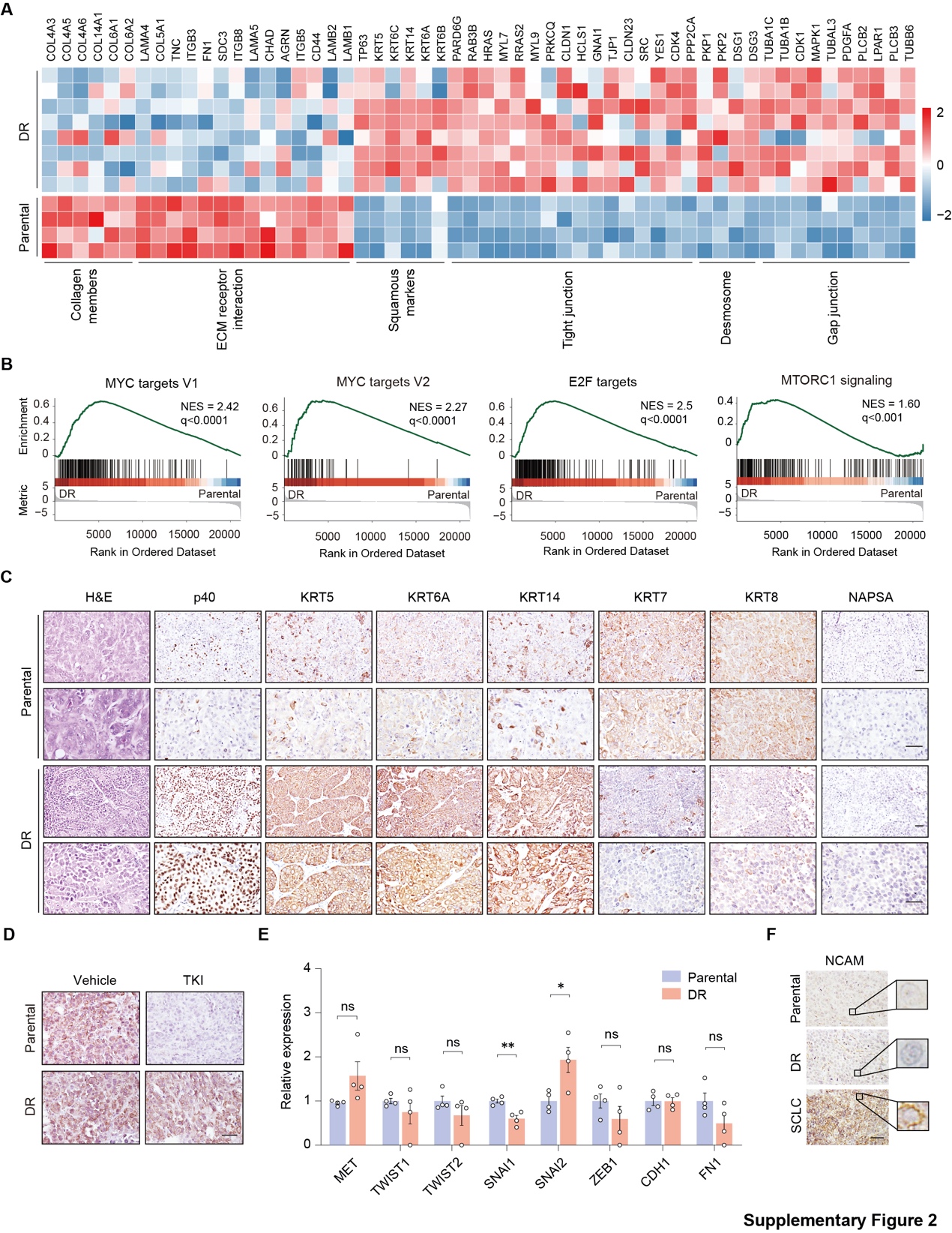


**Supplementary Figure 2. Increased squamous gene signature and decreased adenomatous associated gene signature with TKI resistance development**

(**A**) Heatmap of differentially expressed gene signature in parental (n = 4) and DR (n = 8) tumors. (**B**) GSEA enrichment plots of pathways enriched in parental (n = 4) or DR (n = 8) tumors. (**C**) Representative H&E and immunostaining in parental and OSI-resistant tumors. Scale bar, 50 μm. (**D**) Representative immunostaining of phosphorylated EGFR (Y1086) in parental and DR tumors treated with osimertinib. (**E**) PCR quantitation of mRNA levels in parental and DR tumors. Data are shown as mean ± SEM. *p < 0.05; Statistical significance was calculated by two-tailed unpaired Student’s *t*-test. (**F**) Representative immunostaining of NCAM in parental, DR and SCLC tumors. Scale bar, 50 μm.


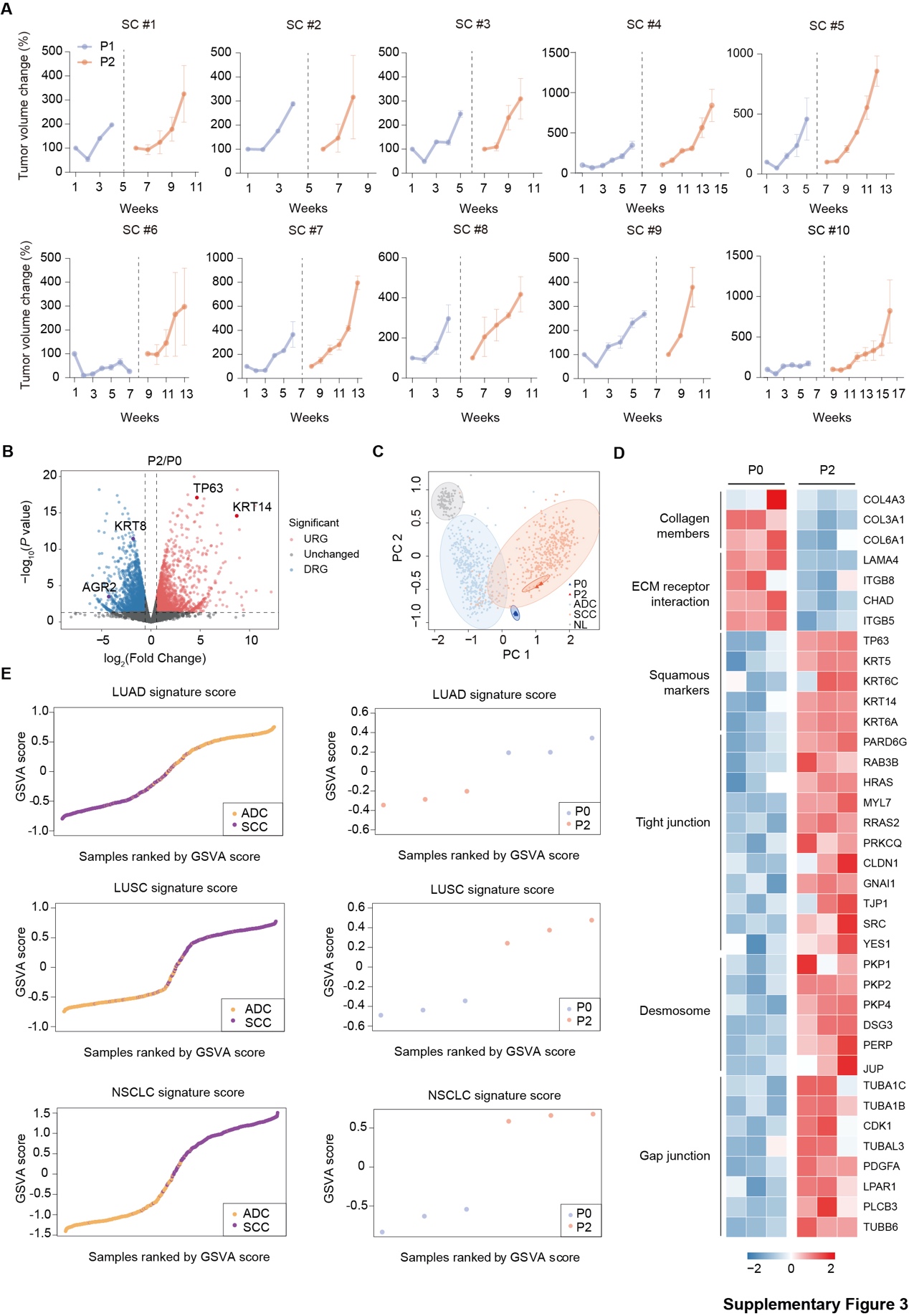


**Supplementary Figure 3. Squamous transition occurs concomitantly with TKI resistance development in single clone-derived PC9 tumors**

(**A**) Relative tumor growth of PC9 single clone tumors under TKI (gefitinib) treatments. (**B**) Volcano plots showing differential expressed genes in single clone passage 0 (P0) and passage 2 (P2) tumors (n = 3 per group). (**C**) Principal-component analysis (PCA) of RNA-seq data from P0 tumors (n = 3), P2 tumors (n = 3), human ADC, SCC and normal lung samples from TCGA. (**D**) Heatmap of differentially expressed gene signature in single clone derived P0 (n = 3) and P2 (n = 3) tumors. (**E**) GSVA scores of TCGA ADC, SCC, NSCLC (left lane) and single clone derived P0 and P2 tumors (right lane) using TCGA ADC, SCC and NSCLC signature.


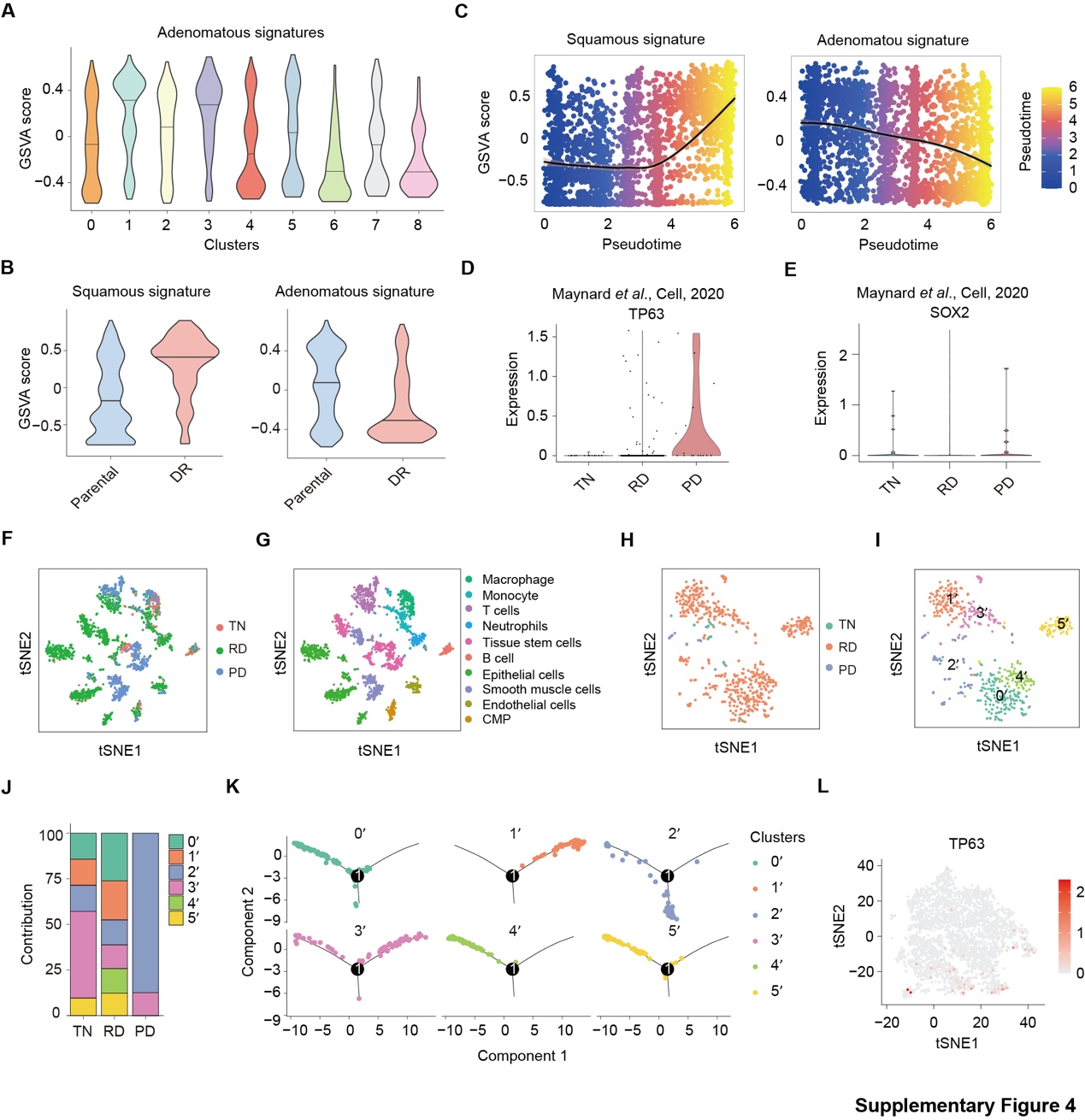


**Supplementary Figure 4. ScRNA-seq delineates squamous transition process with acquisition of TKI resistance**

(**A**) Violin plots depicting the GSVA score of adenomatous signature in indicated clusters. (**B**) GSVA score of squamous signature (left) and adenomatous signature (right) in parental and DR tumors. (**C**) GSVA score of squamous signature (left) and adenomatous signature (right) during pseudotime ordering. (**D**) Violin plots depicting the expression of TP63 in TKI treatment naïve (TN), residual disease (RD) and progressive disease (PD) samples. (**E**) Violin plots depicting the expression of SOX2 in TN, RD and PD samples. (**F**) t-SNE visualization of all cells labeled with original sample source for TN, RD and PD from the Maynard et al. scRNA-seq data. Number of cells (TN: n=246, RD: n=1497, PD: n=833). (**G**) t-SNE visualization of cell types. (**H**) t-SNE visualization of epithelial cells in TN, RD and PD samples. Number of cells (TN: n=21, RD: n=555, PD: n=16). (**I**) t-SNE visualization of 6 clusters in TN, RD and PD samples. (**J**) Bar plot showing distribution of each cluster in TN, RD and PD samples. (**K**) Distribution of each cluster during pseudotime ordering. (**L**) t-SNE visualization of TP63 positive cells in parental tumor cells.


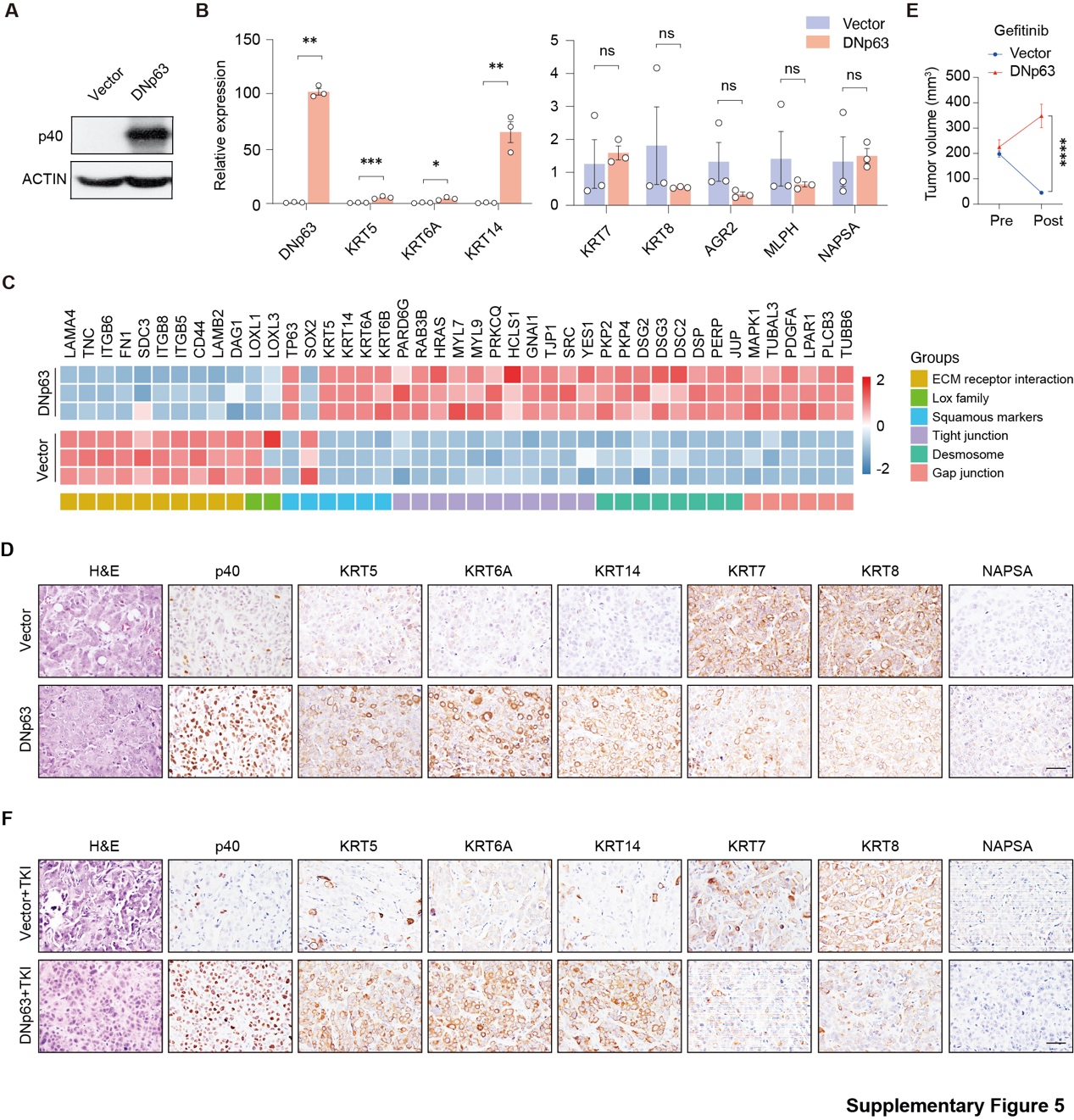


**Supplementary Figure 5. DNp63 overexpression promotes AST and TKI resistance**

(**A**) Immunoblotting detection of p40 of indicated PC9 cells. (**B**) PCR quantitation of mRNA levels in parental tumors with or without DNp63 overexpression. Data are shown as mean ± SEM. *p < 0.05; **p < 0.01; ***p < 0.001; Statistical significance was calculated by two-tailed unpaired Student’s *t*-test. (**C**) Heatmap of differentially expressed gene signature in PC9 cells with or without DNp63 overexpression. (**D**) Representative H&E and immunostaining in treatment naïve PC9 tumors with or without DNp63 overexpression. Scale bar, 50 μm. (**E**) Tumor volumes of PC9 tumors with or without DNp63 overexpression pre- and post- 1 week of TKI (gefitinib) treatments. (**F**) Representative H&E and immunostaining in TKI (gefitinib) treated PC9 tumors with or without DNp63 overexpression. Scale bar, 50 μm.


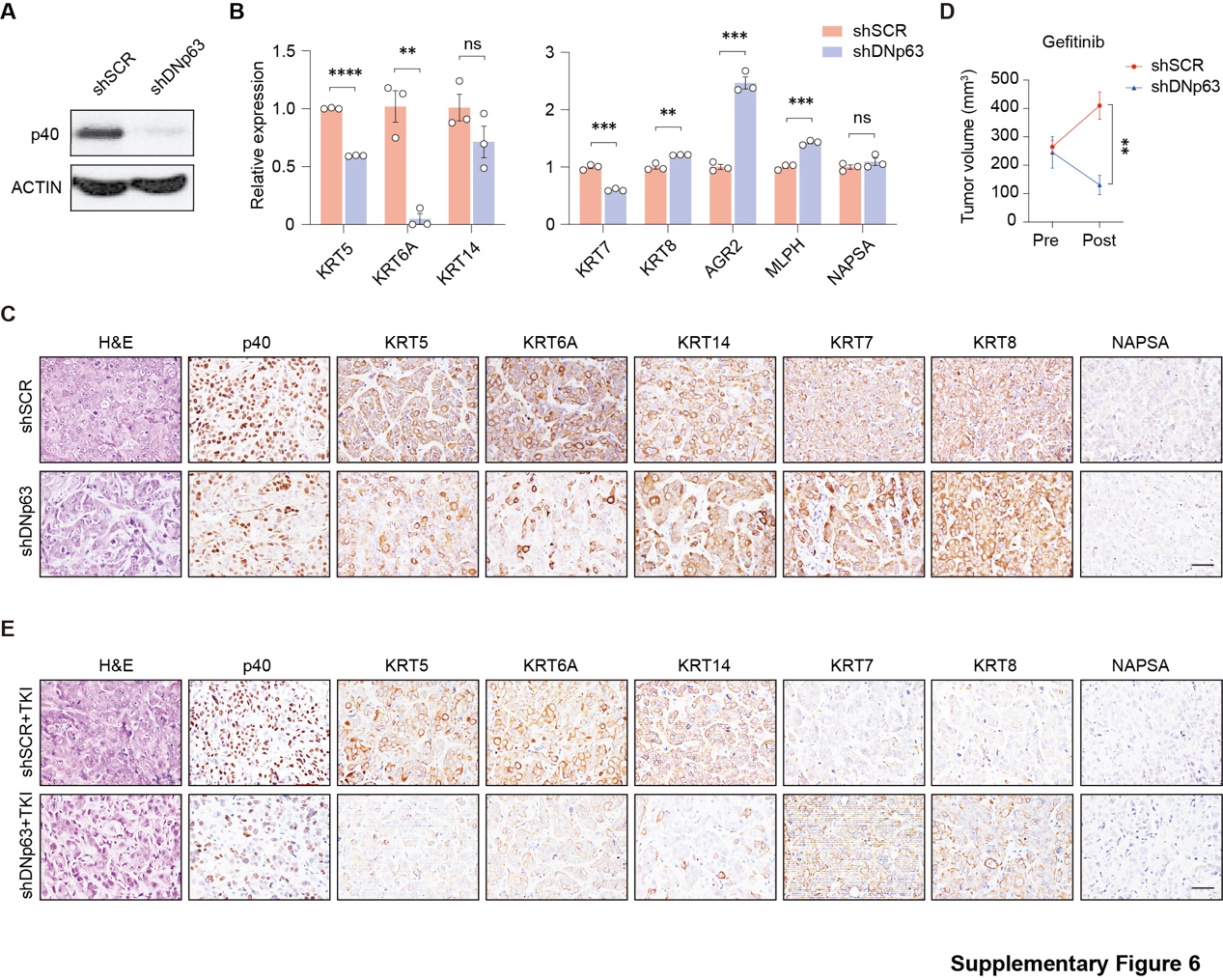


**Supplementary Figure 6. DNp63 knockdown reverses AST and TKI resistance**

(**A**) Immunoblotting detection of p40 in DR cell with or without DNp63 knockdown. (**B**) PCR quantitation of mRNA levels in DR cell with or without DNp63 knockdown. Data are shown as mean ± SEM. **p < 0.01; ***p < 0.001; ****p<0.0001; Statistical significance was calculated by two-tailed unpaired Student’s *t*-test. (**C**) Representative H&E and immunostaining in treatment naïve DR tumors with or without DNp63 knockdown. Scale bar, 50 μm. (**D**) Tumor volumes of DR tumors with or without DNp63 knockdown pre- and post- 1 week of TKI (gefitinib) treatments. (**E**) Representative H&E and immunostaining in TKI (gefitinib) treated DR tumors with or without DNp63 knockdown. Scale bar, 50 μm.


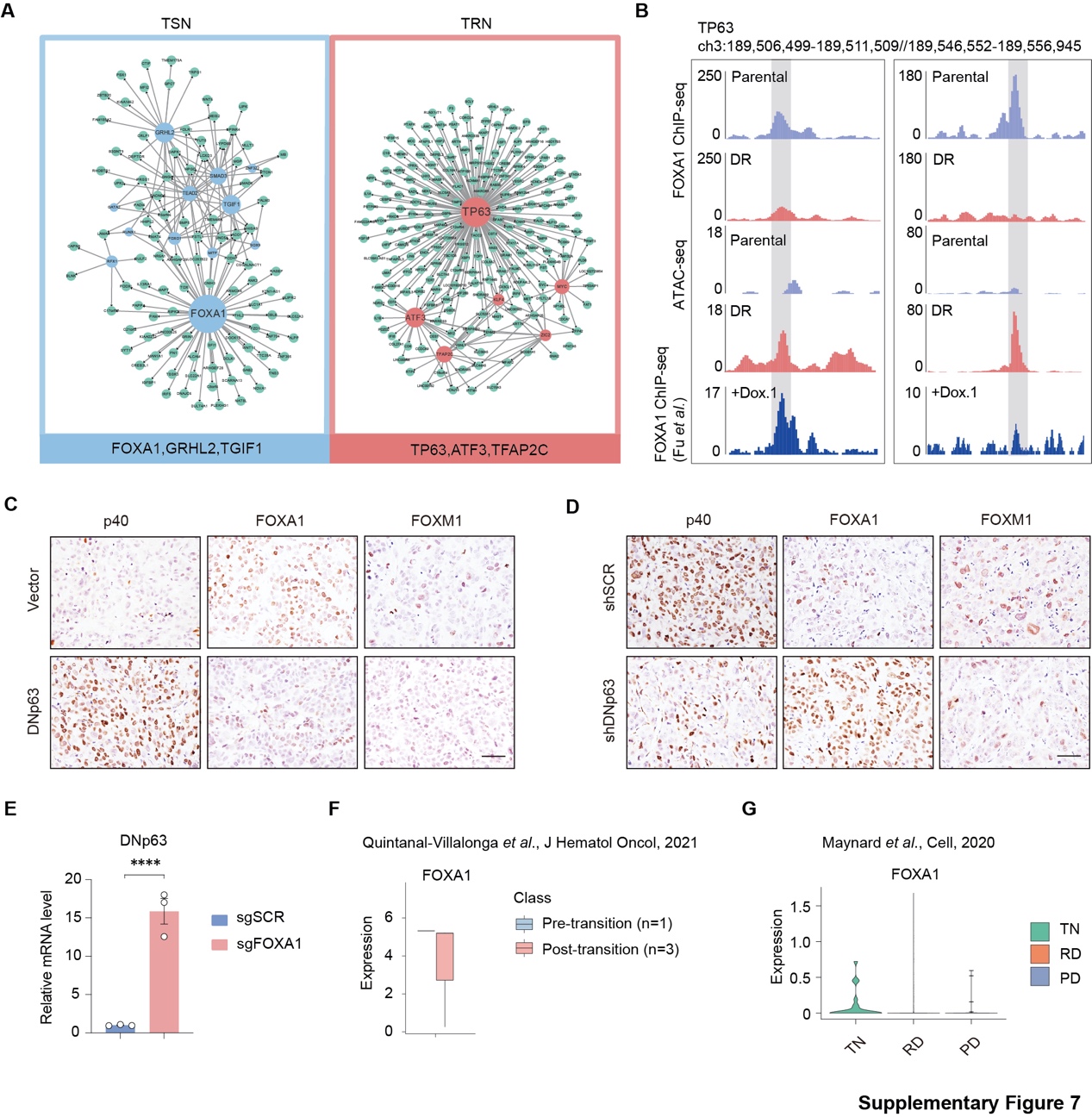


**Supplementary Figure 7. Establishment of TF-chromatin transcriptional regulatory network**

(**A**) TKI sensitive and TKI resistant TF-chromatin transcriptional regulatory networks.

(**B**) FOXA1 ChIP-seq and ATAC-seq tracks in *TP63* loci of parental PC9 cells and DR cells. The FOXA1 ChIP-seq tracks in *TP63* loci of FOXA1-overexpressing MCF7L cells were from Fu *et al*. study [36]. (**C**) Representative immunostaining in control and DNp63-overexpressing tumors. Scale bar, 50 μm. (**D**) Representative immunostaining in control and shDNp63 tumors. Scale bar, 50 μm. (**E**) PCR quantitation of DNp63 mRNA levels in PC9 cells with or without FOXA1 knockout. (**F**) Gene expression of FOXA1 in pre-transition (n = 1) and post-transition (n = 3) samples using the Villalonga *et al*. RNA-seq data. (**G**) Violin plots depicting the expression of FOXA1 in TN, RD and PD samples using the Maynard *et al*. scRNA-seq data.


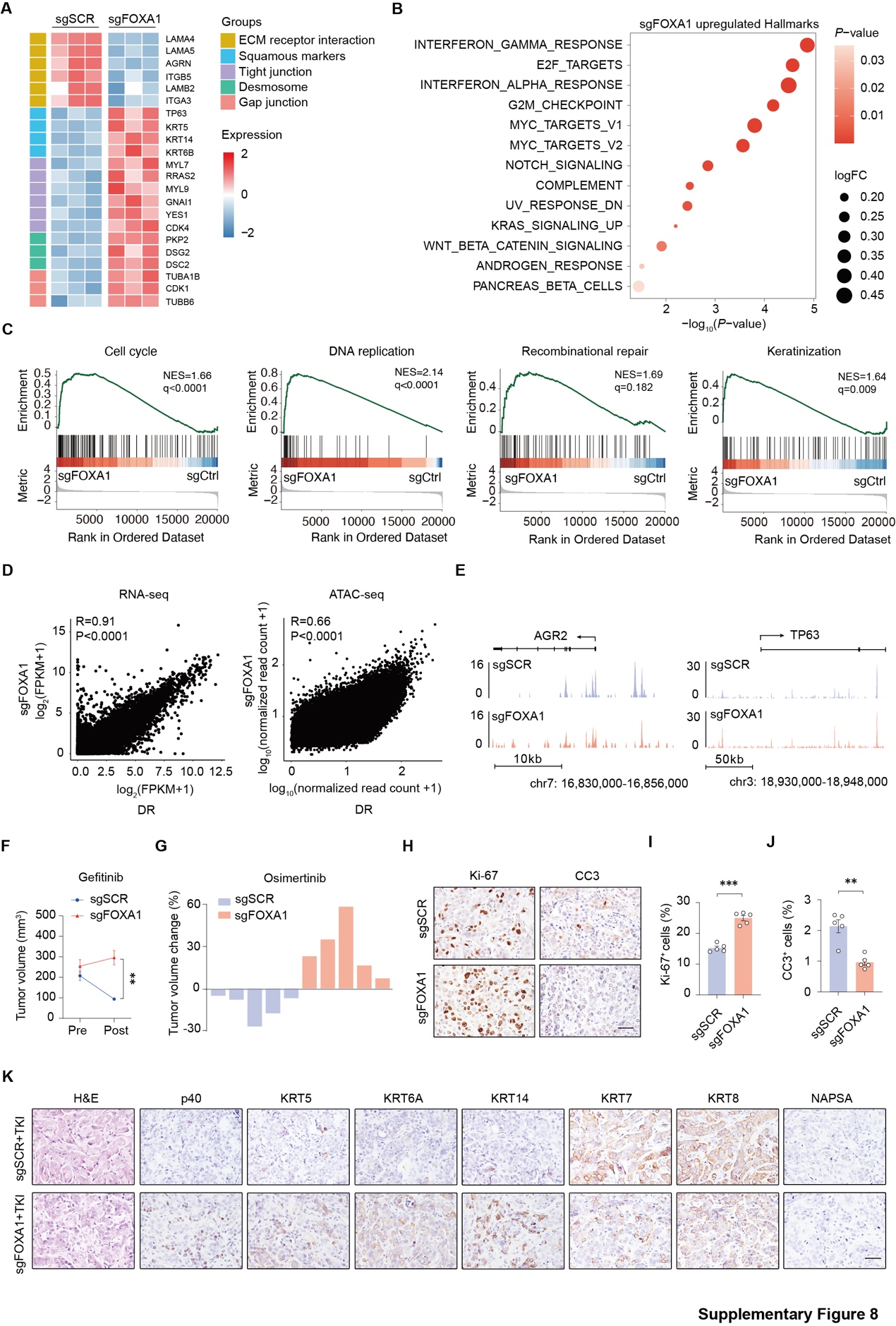


**Supplementary Figure 8. FOXA1 knockout partially promotes AST and TKI resistance**

(**A**) Heatmap of differentially expressed gene signature in control and sgFOXA1 cells.

(**B**) Representative gene pathways of control and sgFOXA1 cells. Pathway enrichment scores were calculated by GSVA and differential analysis was performed by limma. (**C**) GSEA analysis of cell cycle, DNA replication, recombinational repair and keratinization in control and sgFOXA1 cells. (**D**) Scatterplot of gene expression from RNA-seq (left) and read counts from ATAC-seq (right) in control and sgFOXA1 cells.

(**E**) Normalized ATAC-seq profiles at *AGR2*, *TP63* loci in control and sgFOXA1 cells.

(**F**) Tumor volumes of control and sgFOXA1 tumors pre- and post- 1 week of TKI (gefitinib) treatments. (**G**) Tumor volume changes of control and sgFOXA1 tumors after 1 week of TKI (osimertinib) treatments. (**H**, **I** and **J**) Representative immunostaining (**H**) and statistical analysis for Ki-67 (**I**) and CC3 (**J**) in control and sgFOXA1 tumors after 1 week of TKI (osimertinib) treatments. Data are shown as mean ± SEM. **p < 0.01; ***p < 0.001; Statistical significance was calculated by two-tailed unpaired Student’s *t*-test. (**K**) Representative H&E and immunostaining in TKI (gefitinib) treated control and sgFOXA1 tumors. Scale bar, 50 μm.


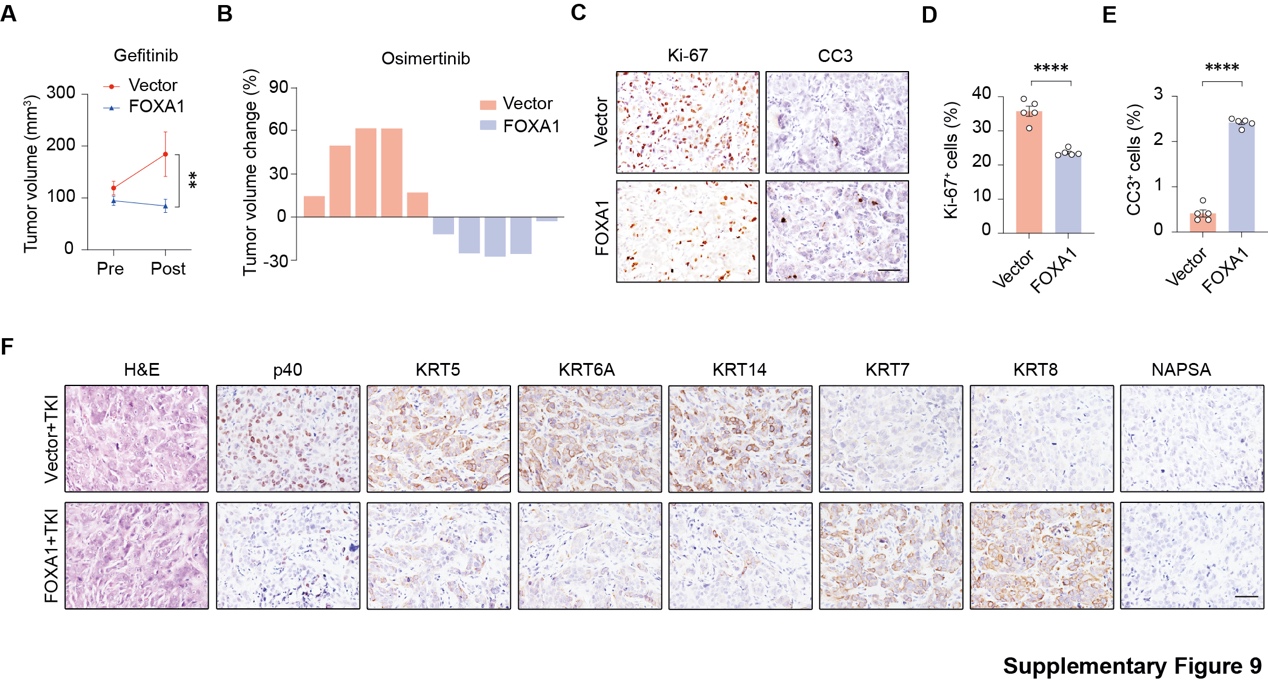


**Supplementary Figure 9. FOXA1 overexpression inhibits AST and restores DR tumor sensitivity to TKI**

(**A**) Tumor volumes of control and FOXA1-overexpressing tumors pre- and post- 1 week of TKI (gefitinib) treatments. (**B**) Tumor volume changes of control and FOXA1-overexpressing tumors after 1 week of TKI (osimertinib) treatments. (**C**, **D** and **E**) Representative immunostaining (**C**) and statistical analysis for Ki-67 (**D**) and CC3 (**E**) in control and FOXA1-overexpressing tumors after 1 week of TKI (osimertinib) treatments. Data are shown as mean ± SEM. **p < 0.01; ***p < 0.001; Statistical significance was calculated by two-tailed unpaired Student’s *t*-test. (**F**) Representative H&E and immunostaining in TKI (gefitinib) treated control and FOXA1-overexpressing tumors. Scale bar, 50 μm.


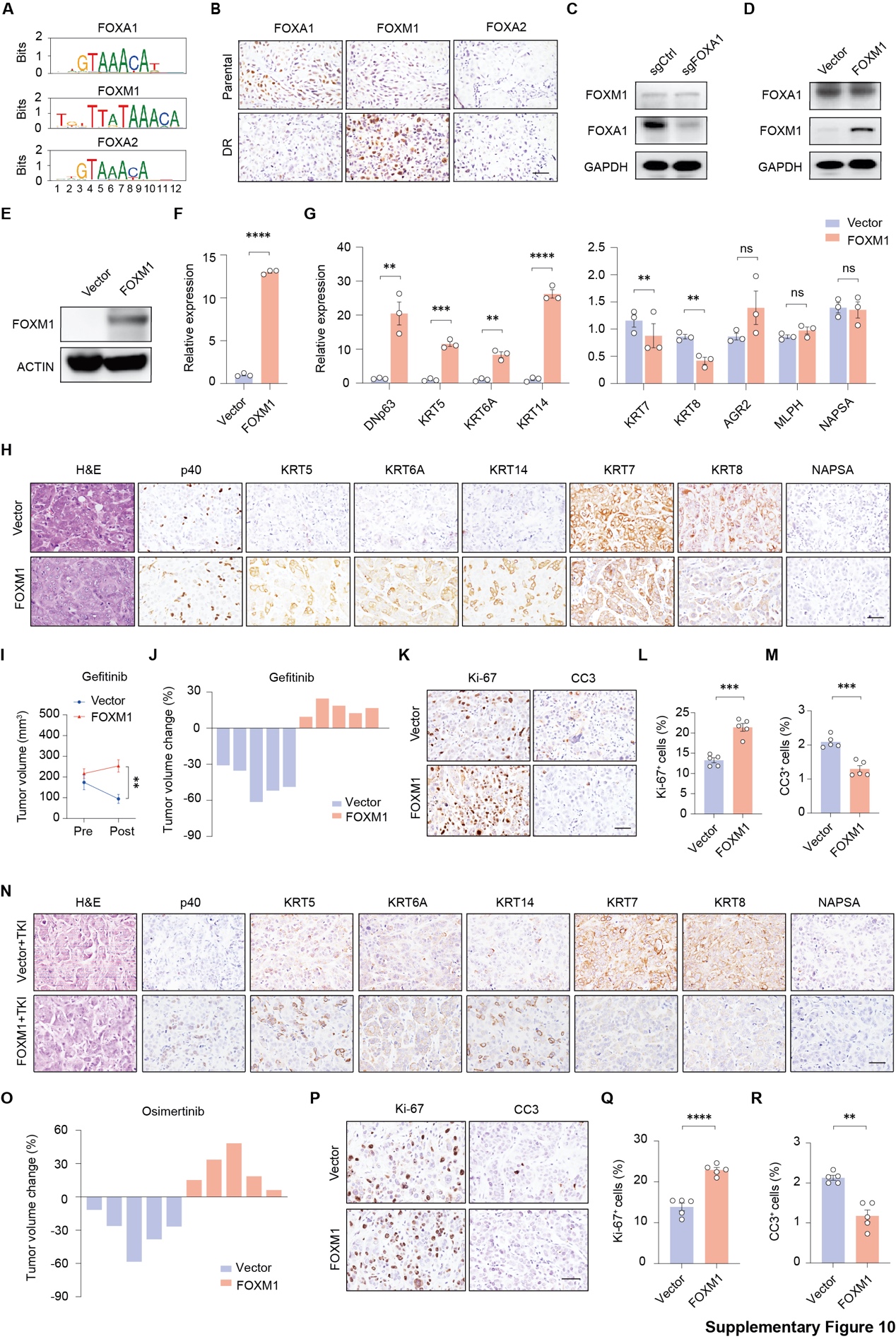


**Supplementary Figure 10. FOXM1 partially induces AST and TKI resistance**

(**A**) Prediction of motifs of FOXA1, FOXM1 and FOXA2 from footprintDB website.

(**B**) Representative immunostaining for FOXA1, FOXM1 and FOXA2 in parental and DR tumors. Scale bar, 50 μm. (**C**) Immunoblotting analysis of FOXM1 in control and sgFOXA1 cells. (**D**) Immunoblotting analysis of FOXA1 in control and FOXM1-overexpressing cells. (**E**) Immunoblotting analysis of FOXM1 in PC9 cells with FOXM1 overexpression. (**F**) PCR quantitation of mRNA levels in PC9 cells with FOXM1 overexpression. (**G**) PCR quantitation of mRNA levels in control (n = 3) and FOXM1-overexpressing tumors (n = 3). Data are shown as mean ± SEM. *p < 0.05; **p < 0.01; ***p < 0.001; ****p<0.0001; Statistical significance was calculated by two-tailed unpaired Student’s *t*-test. (**H**) Representative H&E and immunostaining in control and FOXM1-overexpressing tumors. Scale bar, 50 μm. (**I**) Tumor volumes of control and FOXM1-overexpressing tumors pre- and post- 1 week of TKI (gefitinib) treatments. (**J**) Tumor volume changes in control and FOXM1-overexpressing tumors after 1 week of TKI (gefitinib) treatments. (**K**, **L** and **M**) Representative immunostaining (**K**) and statistical analysis for Ki-67 (**L**) and CC3 (**M**) in control and FOXM1-overexpressing tumors after 1 week of TKI (gefitinib) treatments. Data are shown as mean ± SEM. **p < 0.01; Statistical significance was calculated by two-tailed unpaired Student’s *t*-test. (**N**) Representative H&E and immunostaining in TKI (gefitinib) treated control and FOXM1-overexpressing tumors. Scale bar, 50 μm. (**O**) Tumor volume changes of control and FOXM1-overexpressing tumors after 1 week of TKI (osimertinib) treatments. (**P**, **Q** and **R**) Representative immunostaining (**P**) and statistical analysis for Ki-67 (**Q**) and CC3 (**R**) in control and FOXM1-overexpressing tumors after 1 week of TKI (osimertinib) treatments. Data are shown as mean ± SEM. **p < 0.01; 0.001; Statistical significance was calculated by two-tailed unpaired Student’s *t*-test.


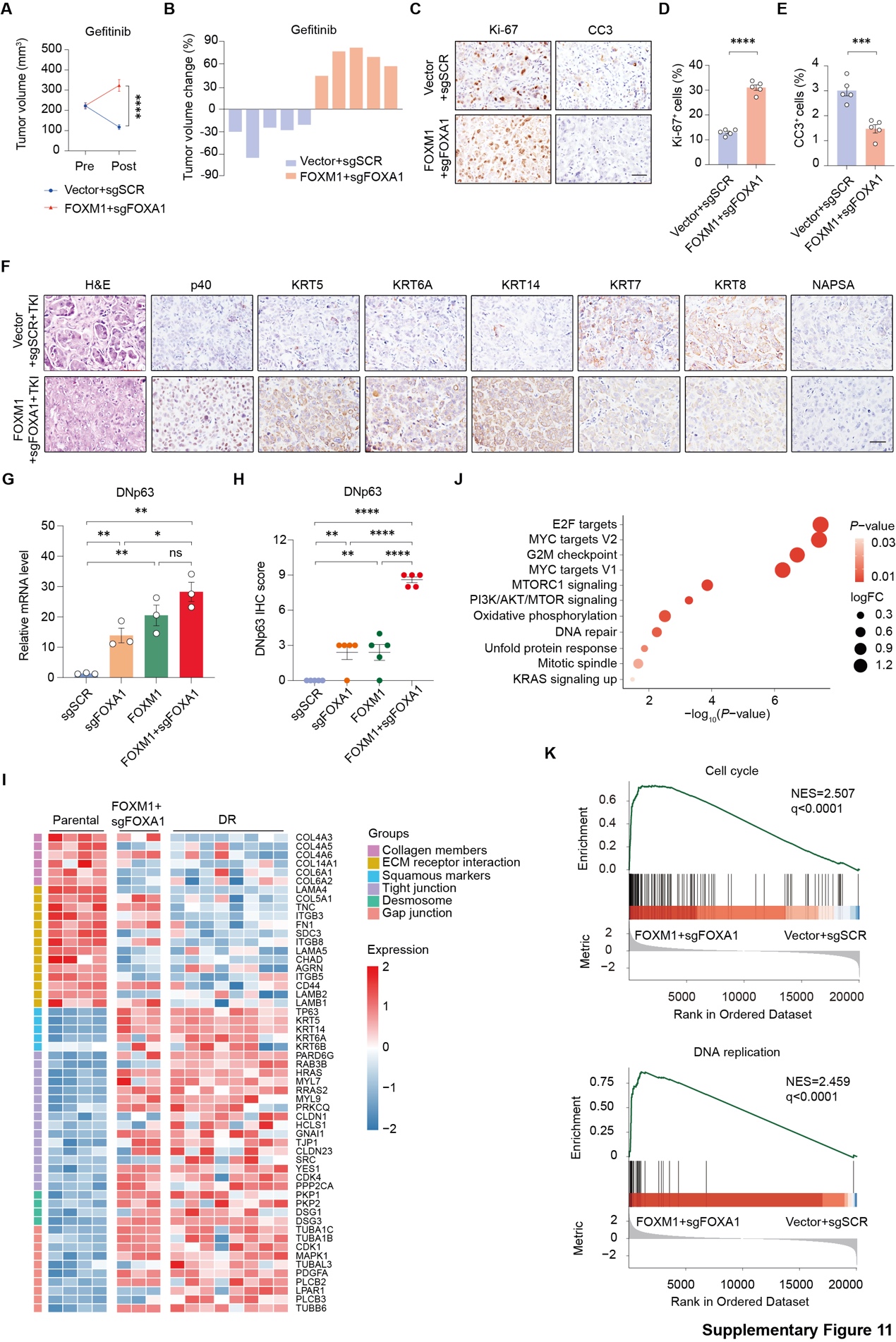


**Supplementary Figure 11. Concomitant FOXM1 overexpression and FOXA1 knockout fully recapitulates AST and TKI resistance**

(**A**) Tumor volumes of control and FOXM1+sgFOXA1 tumors pre- and post- 1 week of TKI (gefitinib) treatments. (**B**) Tumor volume changes of control and FOXM1+sgFOXA1 tumors after 1 week of TKI (gefitinib) treatments. (**C**, **D** and **E**) Representative immunostaining (**C**) and statistical analysis of Ki-67 (**D**) and CC3 (**E**) in control and FOXM1+sgFOXA1 tumors after 1 week of TKI (gefitinib) treatments. Data are shown as mean ± SEM. **p < 0.01; ****p < 0.0001; Statistical significance was calculated by two-tailed unpaired Student’s *t*-test. (**F**) Representative H&E and immunostaining in TKI (gefitinib) treated control and FOXM1+sgFOXA1 tumors. Scale bar, 50 μm. (**G**) PCR quantitation of DNp63 mRNA levels in control, sgFOXA1, FOXM1, FOXM1+sgFOXA1 tumors. (**H**) IHC score of DNp63 in control, sgFOXA1, FOXM1, FOXM1+sgFOXA1 tumors. (**I**) Heatmap of differentially expressed gene signature in parental tumors (n=4), FOXM1+sgFOXA1 cells (n=3) and DR tumors (n=8). (**J**) Representative gene pathways of control and FOXM1+sgFOXA1 cells. Pathway enrichment scores were calculated by GSVA and differential analysis was performed by limma. (**K**) GSEA enrichment plots of cell cycle and DNA replication in control and FOXM1+sgFOXA1 cells.


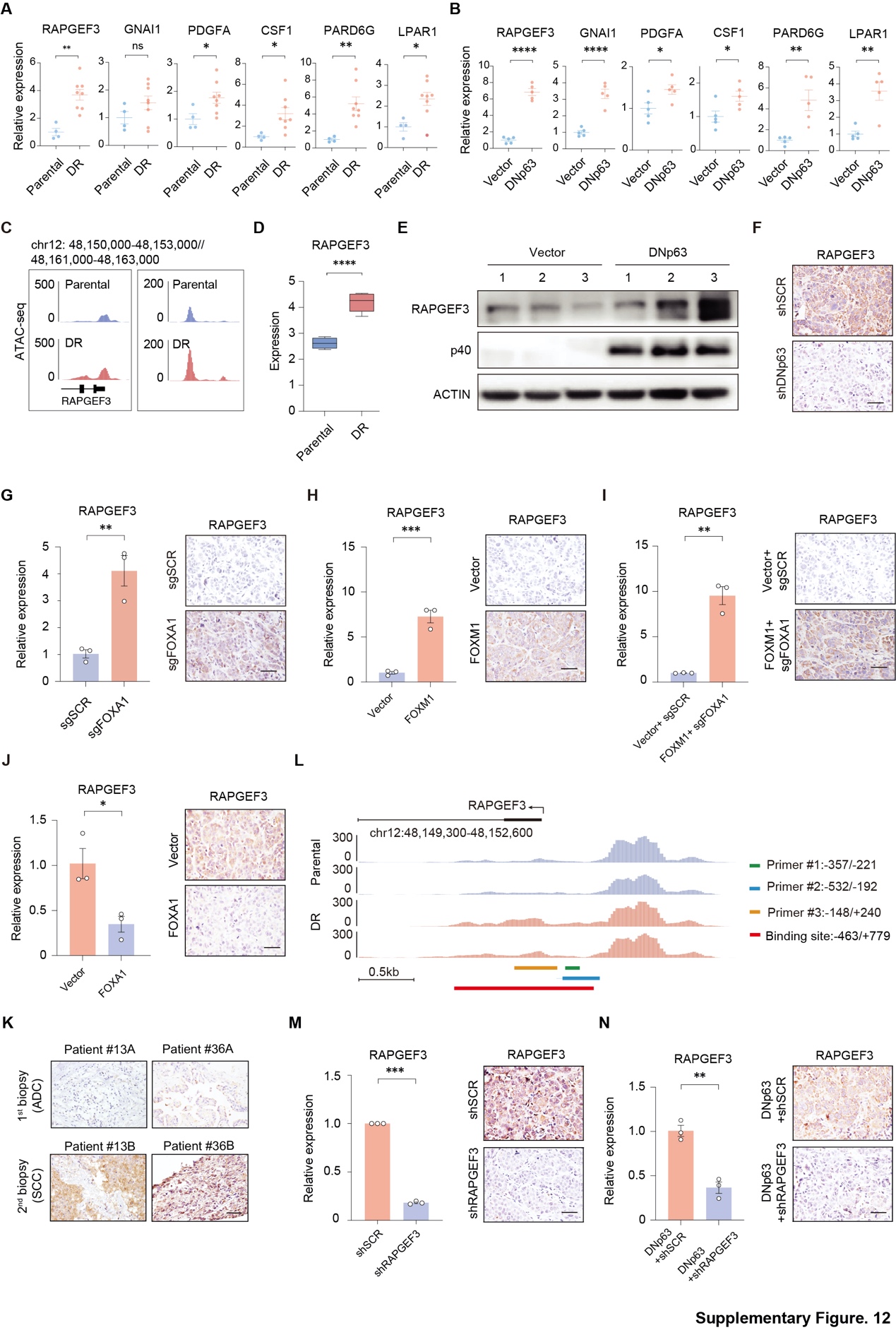


**Supplementary Figure 12. RAPGEF3 acts as potential target in squamous transitioned tumors**

(**A**) PCR quantitation of mRNA levels in parental (n = 5) and DR tumors (n = 5). (**B**) PCR quantitation of mRNA levels in control (n = 5) and DNp63-overexpressing tumors (n = 5). (**C**) ATAC-seq tracks in *RAPGEF3* loci in parental and DR cells. (**D**) RAPGEF3 expression in parental (n=4) and DR (n=8) tumors based on RNA-seq data. (**E**) Immunoblotting analysis of RAPGEF3 and p40 in control and DNp63-overexpressing tumors. (**F**) Representative immunostaining for RAPGEF3 in control and shDNp63 tumors. Scale bar, 50 μm. (**G**) PCR quantitation of mRNA levels (left) and immunostaining (right) of RAPGEF3 in control and sgFOXA1 tumors. (**H**) PCR quantitation of mRNA levels (left) and immunostaining (right) of RAPGEF3 in control and FOXM1-overexpressing tumors. (**I**) PCR quantitation of mRNA levels (left) and immunostaining (right) of RAPGEF3 in control and FOXM1+sgFOXA1 tumors. (**J**) PCR quantitation of mRNA levels (left) and immunostaining (right) of RAPGEF3 in DR control and FOXA1-overexpressing tumors. (**K**) Representative immunostaining staining of RAPGEF3 in two paired human EGFR-mutant lung cancer specimens experiencing squamous transition after EGFR TKI failure (pre- vs. post-transition). Scale bar, 50 μm. (**L**) Potential DNp63 binding loci and primers loci on promoter region of *RAPGEF3* gene. (**M**) PCR quantitation of mRNA levels (left) and immunostaining (right) of RAPGEF3 in DR tumors with or without RAPGEF3 knockdown. (**N**) PCR quantitation of mRNA levels (left) and immunostaining (right) of RAPGEF3 in DNp63-overexpressing tumors with or without RAPGEF3 knockdown. Data are shown as mean ± SEM. *p < 0.05; **p < 0.01; ***p < 0.001; ****p < 0.0001; Statistical significance was calculated by two-tailed unpaired Student’s t-test.

**
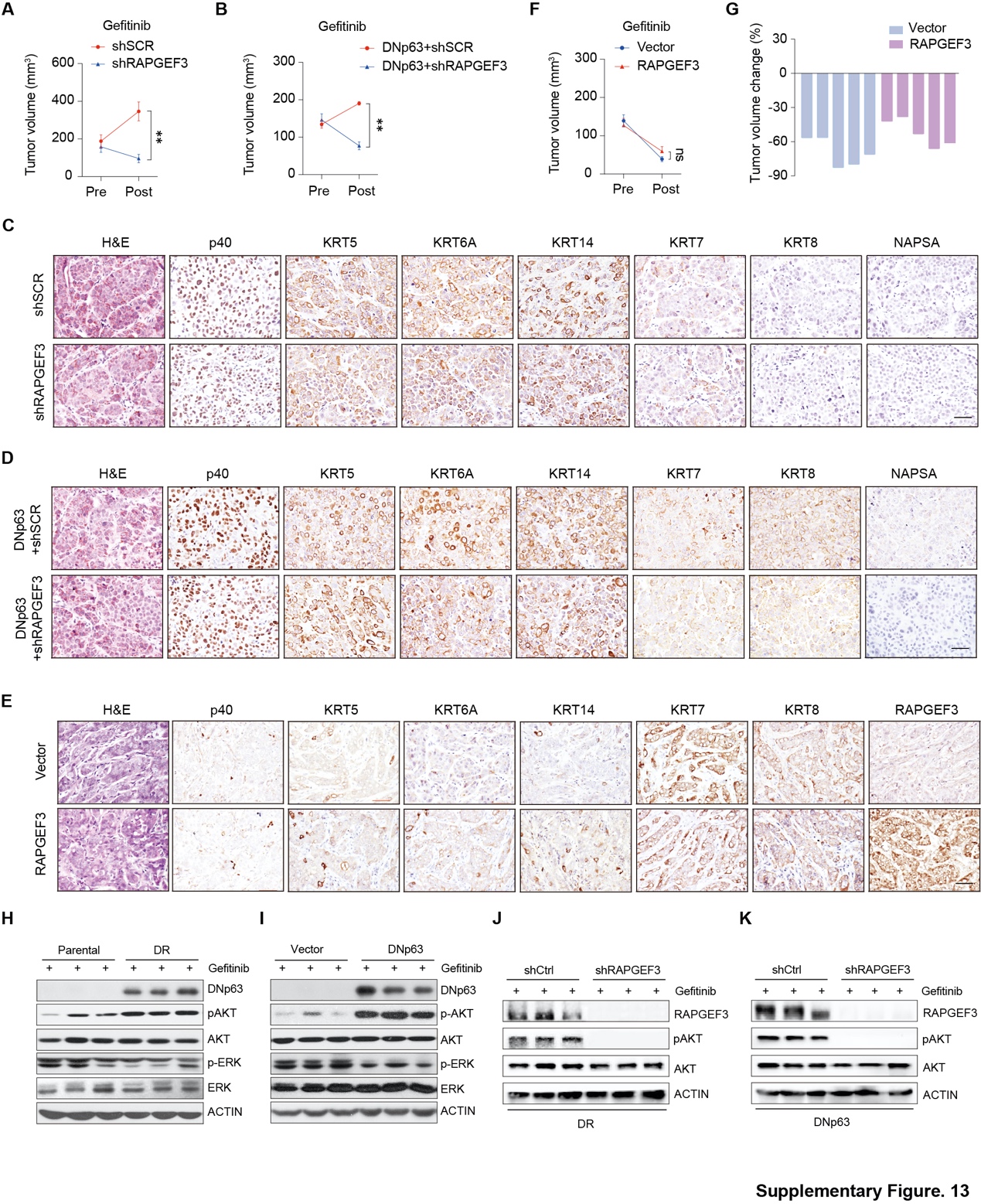
**

**Supplementary Figure 13. RAPGEF3 knockdown restores EGFR TKI sensitivity**

(**A**) Tumor volumes of DR tumors with or without RAPGEF3 knockdown pre- and post- 1 week of TKI (gefitinib) treatments. (**B**) Tumor volumes of DNp63-overexpressing tumors with or without RAPGEF3 knockdown pre- and post- 1 week of TKI (gefitinib) treatments. (**C**) Representative H&E and immunostaining in DR tumors with or without RAPGEF3 knockdown. Scale bar, 50 μm. (**D**) Representative H&E and immunostaining in DNp63-overexpressing tumors with or without RAPGEF3 knockdown. Scale bar, 50 μm. (**E**) Representative H&E and immunostaining in PC9 control and RAPGEF3-overexpressing tumors. Scale bar, 50 μm. (**F**) Tumor volumes of PC9 control and RAPGEF3-overexpressing tumors pre- and post- 1 week of TKI (gefitinib) treatments. (**G**) Tumor volume changes of PC9 control and RAPGEF3-overexpressing tumors after 1 week of TKI (gefitinib) treatments. (**H**) Immunoblotting analysis of EGFR downstream pathways in gefitinib treated PC9 parental and DR tumors. (**I**) Immunoblotting analysis of EGFR downstream pathways in gefitinib treated control and DNp63-overexpressing tumors. (**J**) Immunoblotting analysis of phosphorylated AKT levels in DR tumors with or without RAPGEF3 knockdown after 1 week of TKI (gefitinib) treatments. (**K**) Immunoblotting analysis of phosphorylated AKT levels in DNp63-overexpressing tumors with or without RAPGEF3 knockdown after 1 week of TKI (gefitinib) treatments.


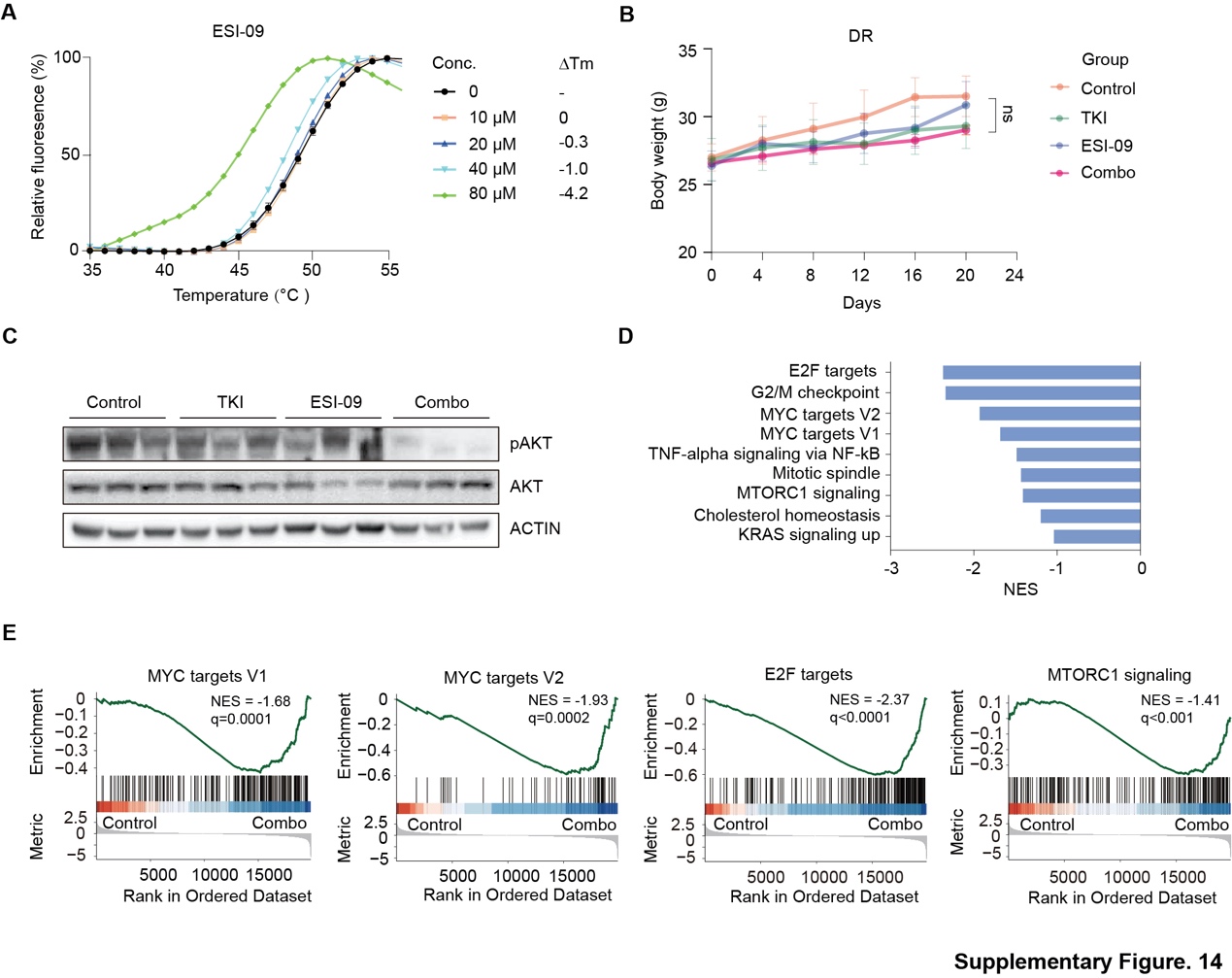


**Supplementary Figure 14. Combined inhibition of EGFR and RAPGEF3 restores EGFR TKI sensitivity in DR tumor**

(**A**) Thermal-induced protein stability of RAPGEF3 in the presence of various ESI-09 concentrations using thermal shift assay. ΔTm: change of thermal melting temperature. (**B**) Body weight changes of DR tumor-bearing mice treated with control, TKI (gefitinib), ESI-09, or combined TKI (gefitinib) +ESI-09. (**C**) Immunoblotting analysis of phosphorylated AKT levels in the DR tumors treated as in (B). (**D**) Representative enriched pathways comparing combined TKI (gefitinib) +ESI-09 versus control tumors. Pathway enrichment scores were calculated by GSVA and differential analysis was performed by limma. (**E**) GSEA enrichment plots of indicated pathways in control and combined treatment tumors.


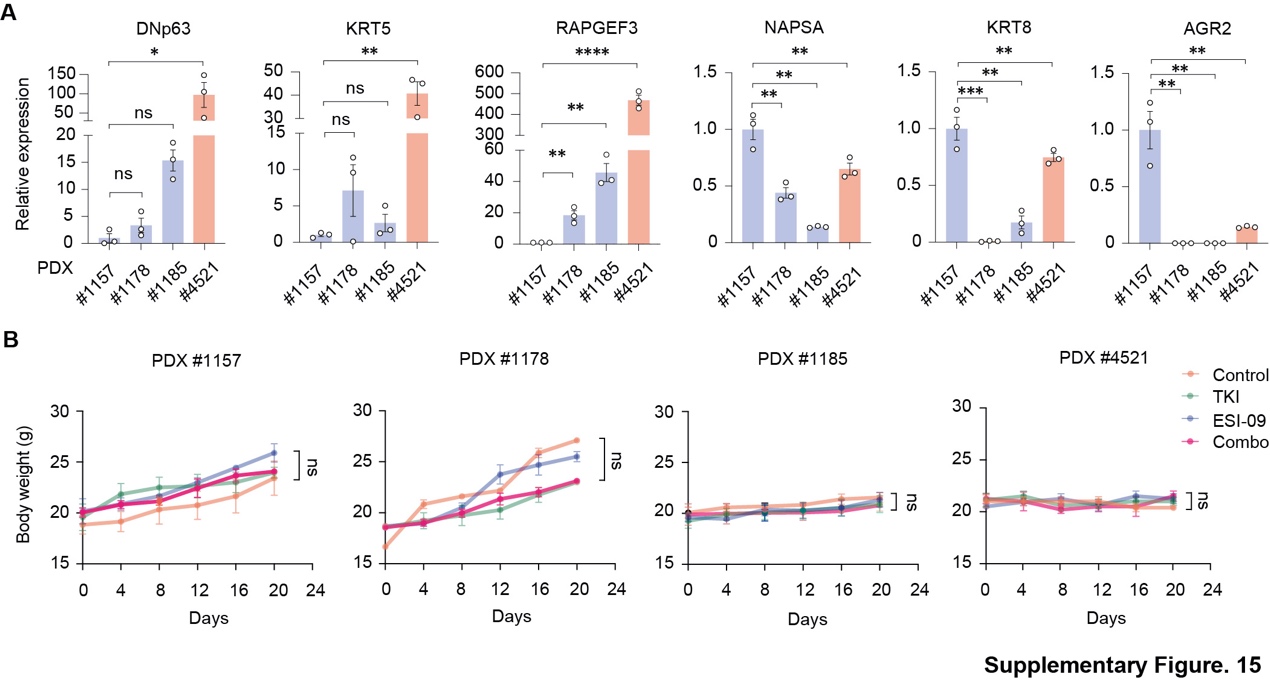


**Supplementary Figure 15. Combined treatments with RAPGEF3 and EGFR inhibitors show no increased systemic toxicity in PDX models**

(**A**) PCR quantitation of mRNA levels of indicated genes in the four PDX models.

(**B**) Body weight changes of PDX models treated with control, TKI, ESI-09, or combined TKI+ESI-09.

**Supplementary Tables**

**Table S1. Gene list of related pathways in Fig. 7B**

| KEGG pathway | Genes |
| --- | --- |
| RAP1 signaling | GNAI1, PDGFA, CSF1, PARD6G, LPAR1, RAPGEF3 |
| cAMP signaling pathway | ADRB2, ATP1B3, GNAI1, RAPGEF3, HCAR3 |
| Adrenergic signaling in cardiomyocytes | ADRB2, ATP1B3, GNAI1, RAPGEF3 |
| p53 signaling pathway | CD82, SFN, GADD45A |
| Inositol phosphate metabolism | INPP1, PIP5KL1, PLCH2 |
| cGMP-PKG signaling pathway | ADRB2, ATP1B3, RGS2, GNAI1 |
| ECM-receptor interaction | SDC1, LAMA3, LAMC2 |

**Table S2. Oligos used in this study**

| Gene name | Sequences |  |  |
| --- | --- | --- | --- |
| hDNp63-F | GGAAAACAATGCCCAGACTC |  |  |
| hDNp63-R | GTGGAATACGTCCAGGTGGC |  |  |
| hSOX2-F | TACAGCATGTCCTACTCGCAG |  |  |
| hSOX2-R | GAGGAAGAGGTAACCACAGGG |  |  |
| hCK14-F | CATGAGTGTGGAAGCCGACAT |  |  |
| hCK14-R | GCCTCTCAGGGCATTCATCTC |  |  |
| hCK5-F | CGAGCAGTACATCAACAACCTCAG |  |  |
| hCK5-R | GGCAGCATCTACATCCTTCTTCAG |  |  |
| KRT6A-F | TGGGTGTGATCTCACTGTTGG |  |  |
| KRT6A-R | GACTAGGAATCAGGCTCGGG |  |  |
| hCK7-F | CTCCGGAATACCCGGAATGAG |  |  |
| hCK7-R | ATCACAGAGATATTCACGGCTCC |  |  |
| hCK8-F | TCCTCAGGCAGCTATATGAAGAG |  |  |
| hCK8-R | GGTTGGCAATATCCTCGTACTGT |  |  |
| hNAPSA-F | GGAGCCTGAGGAGGCC |  |  |
| hNAPSA-R | GGACTTGGGATTAATGCG |  |  |
| hMET-F | GGTTCACTGCATATTCTCCCC |  |  |
| hMET-R | ACCATCTTTCGTTTCCTTTAGCC |  |  |
| hCDH1-F | ATTTTTCCCTCGACACCCGAT |  |  |
| hCDH1-R | TCCCAGGCGTAGACCAAGA |  |  |
| hTWIST1-F | GTCCGCAGTCTTACGAGGAG |  |  |
| hTWIST1-R | GCTTGAGGGTCTGAATCTTGCT |  |  |
| hTWIST2-F | GCAAGAAGTCGAGCGAAGAT |  |  |
| hTWIST2-R | GCTCTGCAGCTCCTCGAA |  |  |
| hSNAI1-F | CTGCCCTGCGTCTGCGGAAC |  |  |
| hSNAI1-R | GGAGCGGTCAGCGAAGGCAC |  |  |
| hSNAI2-F | CCGCGCTCCTTCCTGGTCAA |  |  |
| hSNAI2-R | TGGAGCAGCGGTAGTCCACACA | |  |
| hZEB1-R  hZEB1-F | GGTCCTCTTCAGGTGCCTCAG  AGCAGTGAAAGAGAAGGGAATGC | |  |
| hZEB2-F | CAAGAGGCGCAAACAAGCC | |  |
| hZEB2-R | GGTTGGCAATACCGTCATCC | |  |
| hFN1-F | CGGTGGCTGTCAGTCAAAG | |  |
| hFN1-R | AAACCTCGGCTTCCTCCATAA | |  |
| hGANI1-F | GAGATTCAAAACCCCAAACCCG | |  |
| hGANI1-R | AGGTTGCGGTCGATCATCTT | |  |
| hPDGFA-F | CCCTGCCCATTCGGAGGAAGA | |  |
| hPDGFA-R | TTGGCCACCTTGACGCTGCG | |  |
| hRAPGEF3-F | AGTTTCCCACCTCCACGAGGAC | |  |
| hRAPGEF3-R | ACATAAGCCCAGGTGCTGGCTG | |  |
| hPARD6G-F | CGAAACCATGAACCGAAGTT | |  |
| hPARD5G-R | GGCTTATGACGGTCCAGAGA | |  |
| hATP1B3-F | CCCGTAACGAGGAGGTGTTC | |  |
| hATP1B3-R | GGCTCTGGTTGAGGGACTTC | |  |
| hADRB2-F | CGTGTCCTTCTACGTTCCCC | |  |
| hADRB2-R | AAGATCTGCGGAGTCCATGC | |  |
| hLPAR1-F | CTCGGCATAGTTCTGGACCC | |  |
| hLPAR1-R | TTCTCATAGGCCAGCACGTC | |  |
| hCSF1-F | TGTGGTTTGTGGGAAAGCAG | |  |
| hCSF1-R | CTTCAGGCTCCTCTCTCTGG | |  |
| hHCAR3-F | GCCCAACCTCAAATAACCATTCC | |  |
| hHCAR3-R | CTCGATGCAACAGCCCAACT | |  |
| hGAPDH-F | GCCTCAAGATCATCAGCAATGCCT | |  |
| hGAPDH-R | TGTGGTCATGAGTCCTTCCACGAT | |  |
| hFOXM1-R  hFOXM1-F | GGTCCAGTGGCTTAAACACC  AACCTTTCCCTGCACGACAT | | |
| sgFOXA1 F | CACCGGTTGGACGGCGCGTACGCCA | | |
| sgFOXA1 R | AAACTGGCGTACGCGCCGTCCAACC | | |
| sgFOXA1 F PCR | CATGAACAGCATGACTGCG | | |
| sgFOXA1 R PCR | GATGAGCGAGATGTACGAGTAGG | | |
| shRAPGEF3-3 F | CCGGGGCTCAATGAGCGTCTCTTTGCTCGAGCAAAGAGACGCTCATTGAGCCTTTTTG | | |
| shRAPGEF3-3 R | AATTCAAAAAGGCTCAATGAGCGTCTCTTTGCTCGAGCAAAGAGACGCTCATTGAGCC | | |
| shDNp63-2 F | CCGGGAGTGGAATGACTTCAACTTTCTCGAGAAAGTTGAAGTCATTCCACTCTTTTTG | | |
| shDNp63-2 R | AATTCAAAAAGAGTGGAATGACTTCAACTTTCTCGAGAAAGTTGAAGTCATTCCACTC | | |
| Chip RAPGEF3-1 F | CGGAGGGGACACTACTCAAC | | |
| Chip RAPGEF3-1 R | TGGGCAGGTGAAAGGTACTC | | |
| Chip RAPGEF3-2 F | CCTGGGGCAGTTGTACTGTG | | |
| Chip RAPGEF3-2 R | CCTCTAGCTCTGGTACTGGC | | |
| Chip RAPGEF3-3 F | CTCTGGGGGTGTACAGTGGTA | | |
| Chip RAPGEF3-3 R | ACCAAGCAGTGTCCACATGA | | |
| Chip MDM2-F | GGTTGACTCAGCTTTTCCTCTTG | | |
| Chip MDM2-R | GAAAATGCATGGTTTAAATAGCC | | |
